# *Gcm*1 is involved in cell proliferation and fibrosis during kidney regeneration after ischemia–reperfusion injury

**DOI:** 10.1038/s41598-019-44161-y

**Published:** 2019-05-27

**Authors:** Sahoko Kamejima, Norifumi Tatsumi, Akane Anraku, Hideaki Suzuki, Ichiro Ohkido, Takashi Yokoo, Masataka Okabe

**Affiliations:** 1 Department of Anatomy, The Jikei University School of Medicine, Tokyo, Japan; 2 Division of Nephrology and Hypertension, Department of Internal Medicine, The Jikei University School of Medicine, Tokyo, Japan

**Keywords:** Biochemistry, Kidney

## Abstract

In acute kidney injury (AKI), the S3 segment of the proximal tubule is particularly damaged, as it is most vulnerable to ischemia. However, this region is also involved in renal tubular regeneration. To deeply understand the mechanism of the repair process after ischemic injury in AKI, we focused on glial cells missing 1 (*Gcm1*), which is one of the genes expressed in the S3 segment. *Gcm1* is essential for the development of the placenta, and *Gcm1* knockout (KO) is embryonically lethal. Thus, the function of *Gcm1* in the kidney has not been analyzed yet. We analyzed the function of *Gcm1* in the kidney by specifically knocking out *Gcm1* in the kidney. We created an ischemia–reperfusion injury (IRI) model to observe the repair process after AKI. We found that *Gcm1* expression was transiently increased during the recovery phase of IRI. In *Gcm1* conditional KO mice, during the recovery phase of IRI, tubular cell proliferation reduced and *transforming growth factor-β1* expression was downregulated resulting in a reduction in fibrosis. *In vitro*, *Gcm1* overexpression promoted cell proliferation and upregulated *TGF-β1* expression. These findings indicate that *Gcm1* is involved in the mechanisms of fibrosis and cell proliferation after ischemic injury of the kidney.

## Introduction

The kidney is an important organ that removes toxic waste products and excess water from the body in the form of urine, and it is essential for the maintenance of life. Acute kidney injury (AKI) is a state involving a rapid decline in renal function, and it is caused by various factors, such as kidney ischemia, sepsis, and nephrotoxic drugs. Ischemic injury is considered as the most common cause of AKI in clinical practice^1–3^. The progression of AKI has been shown to increase the risk of developing chronic kidney disease (CKD)^4,5^ and cause deterioration of existing CKD^4,6^ and progression to end-stage renal disease^4,7,8^. These changes can increase the number of newly introduced dialysis patients, trigger the development and worsening of cardiovascular diseases, increase the occurrence of complications of metabolic abnormality, and trigger the deterioration of the life prognosis of patients. AKI is known to cause the destruction of renal tubular epithelial cells, and this secondarily causes inflammation and fibrosis of renal parenchyma and rapidly reduces renal function. The injured kidney can recover by proliferation of cells in the proximal tubule^9–11^, which is the main injured site, and recovery can occur within a few days^3,12,13^. However, if the injury is too extensive or does not properly recover owing to insufficient repair of the proximal tubule, the injured kidney may develop chronic fibrotic changes characterized by interstitial enlargement, leukocyte infiltration, and extracellular matrix production. Fibrosis is a general adaptation reaction in the healing process of injured tissue; however, the excessive accumulation of extracellular matrix can destroy normal tissues and cause organ dysfunction, and it is significantly correlated with the extent of renal dysfunction and functional prognosis in CKD^14–17^. It is important to understand the mechanisms that mediate the progression of AKI to CKD to prevent the development of CKD in patients with AKI.

It is known that ischemia/hypoxia is greatly involved in the progression of AKI to CKD. Rarefaction and loss of capillaries around the renal tubules in AKI cause hypoxia. Hypoxia damages renal tubular epithelial cells, activates inflammatory cells and fibroblasts, and eventually causes progression to CKD^18,19^. The S3 segment of the proximal tubule is particularly damaged in AKI^11,20–23^. The S3 segment is most vulnerable to renal ischemia^13,24^ and is involved in renal tubular regeneration^25,26^. It has been reported that the expression of some genes increases in the S3 segment after ischemic injury, resulting in various changes. For example, *Sall1* gene expression makes the kidney vulnerable to ischemia–reperfusion injury (IRI) through a decrease in the expression of heme oxygenase-1^27^, and *IRF*-*1* gene expression promotes inflammation after IRI^28^. Analysis of the genes expressed in the S3 segment is very important to understand the mechanisms of the repair process after ischemic injury and to identify a treatment approach for preventing the progression of AKI to CKD. However, the analysis of other genes expressed in the S3 segment has not yet progressed.

The *glial cells missing 1* (*Gcm1*) gene is one of the genes expressed in the S3 segment^29^, and its function in the kidney is still not entirely known. The Gcm1 protein is a transcription factor involved in development^30^, and in mammals, Gcm1 is mainly expressed in the placenta and kidneys^29^. In the placenta, Gcm1 regulates the expression of the syncytin gene, which promotes the fusion of trophoblasts, is essential for the differentiation of syncytiotrophoblasts, and is a master regulator in complicated labyrinth branching. In previous studies, *Gcm1* knockout (KO) mice could not appropriately form the placental labyrinth, and this condition was embryonically lethal^31,32^. Thus, the function of *Gcm1* in organs other than the placenta, such as the kidney, has not been analyzed yet. The present study analyzed the function of *Gcm1* in the kidney by specifically knocking out *Gcm1* in the kidney. The findings of this study might help better understand fibrosis and cell proliferation after ischemic kidney injury.

## Results

### Gcm1 expression was markedly increased in the recovery phase of IRI

**Figure 1:**
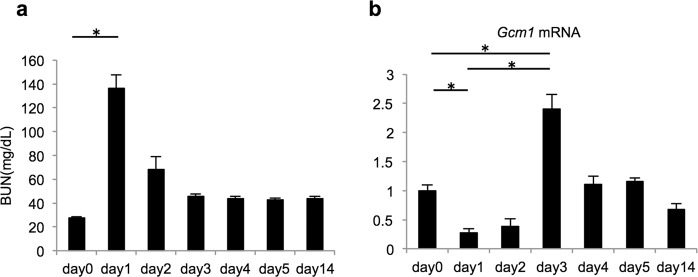
*Gcm1* expression shows a marked increase in the recovery phase of ischemia–reperfusion injury (IRI). (**a**) Blood urea nitrogen (BUN) level is measured as an indicator of renal function in wild-type mice before IRI (day 0) or after IRI (ischemia followed by 1, 2, 3, 4, 5, and 14 days of reperfusion). (**b**) *Gcm1* expression in the kidney is examined using real-time polymerase chain reaction in wild-type mice before IRI (day 0) or after to IRI (ischemia followed by 1, 2, 3, 4, 5, and 14 days of reperfusion). Data are presented as mean ± SEM (*n* = 9). *P < 0.01.

AKI was replicated in an IRI model, and the blood urea nitrogen (BUN) level was measured as an indicator of renal function. The BUN level was significantly higher in day 1 IRI mice than in day 0 mice, and the level decreased after day 1 (Fig. 1a). This result is similar to the finding in a previous report^33^.

Real-time quantitative RT-PCR (qRT-PCR) was performed to assess the change in *Gcm1* expression in the IRI model using kidney samples after IRI. *Gcm1* expression was significantly lower in day 1 IRI mice than in day 0 mice (*P* < 0.01). On the other hand, *Gcm1* expression was significantly higher in day 3 IRI mice than in day 1 IRI mice and day 0 mice (both *P* < 0.01), corresponding to the recovery phase of IRI (Fig. 1b).

### Generation and analysis of Gcm1 conditional KO mice

*Gcm1* expression after IRI was markedly increased in the recovery phase of IRI. This result suggests that *Gcm1* has some function against kidney damage. We generated conditional KO mice by specifically knocking out *Gcm1* in the kidney.

**Figure 2:**
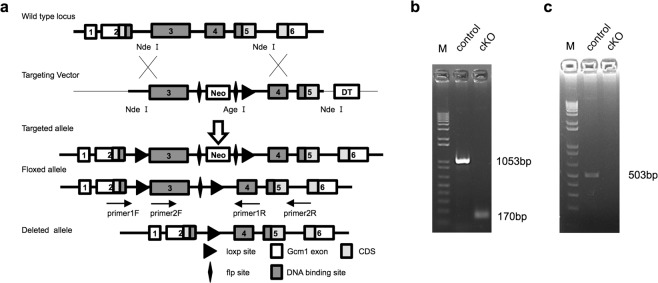
Generation of *Gcm1* conditional knockout (cKO) mice. (**a**) Schema of the construction of alleles and the vector for the mouse *Gcm1* gene. The wild-type allele, targeting vector, flox-neo allele, floxed allele, and deleted allele are shown. The *Gcm1*-floxed mutant mice (Gcm1^*flox*/*flox*^) possess a loxP site before exon 3 and another site upstream of exon 4 of *Gcm1*. (**b**) Genotype analysis in control and cKO mouse kidneys using primer 1 F and 1 R. The left lane shows the finding in control mice (genotype: Gcm1^*flox*/*flox*^), and the right lane shows the finding in cKO mice (genotype: Wt1 ^*GFPCre*/+^; Gcm1^*flox*/*flox*^). (**c**) Real-time polymerase chain reaction analysis of *Gcm1* in control and cKO mouse kidneys using primer 2F and 2R. The left lane shows the finding in control mice, and the right lane shows the finding in cKO mice. M, molecular weight marker.

*Gcm1*, which is a transcriptional regulator, is composed of six exons, and exon 3 primarily codes for a major part of the DNA binding site^34^. We created *Gcm1*-floxed mice (Gcm1^*flox*/*flox*^ mice) with the exon 3 of *Gcm1* flanked by loxP sites to knock out exon 3 in the Cre-LoxP system (Fig. 2a). Since Wt1 is expressed in the metanephric mesenchyme during embryonic kidney development^35^, Gcm1^*flox*/*flox*^ mice were crossed with Wt1^*GFPCre/*+^ mice to create Wt1^*GFPCre/*+^; Gcm1^*flox*/*flox*^ mice which conditionally knock out *Gcm1* in renal tubules. These mice were crossed with Gcm1^*flox*/*flox*^ mice to obtain Gcm1^*flox*/*flox*^ control mice and Wt1^*GFPCre/*+^; Gcm1^*flox*/*flox*^ KO (cKO) mice. Genomic PCR was performed to check whether *Gcm1* was properly knocked out in the kidney. In control mice, a band measuring 1053 bp was observed, and in cKO mice, the region between loxP sites was lost and a band measuring 170 bp was observed (Fig. 2b). Additionally, the finding was confirmed with RT-PCR using cDNA prepared from the kidney. In control mice, a band measuring 503 bp was observed, and in cKO mice, exon 3 disappeared and no band was observed (Fig. 2c). These results confirmed that *Gcm1* was completely knocked out in the kidney. We assessed *Gcm1* expression in the kidneys of mice using *in situ* hybridization. In control mice, *Gcm1* expression was noted in the cortex region, and the strongest staining was observed at the corticomedullary junction where the S3 segment exists^29^ (Fig. S1). On the other hand, in cKO mice, no expression was observed. Thus, it was confirmed that in cKO mice, *Gcm1* was knocked out in the entire cortex region and corticomedullary junction where it is usually expressed.

To assess whether the morphology of the cKO kidney differs from the usual morphology, we observed the morphology of the E18.5 cKO kidney. On hematoxylin and eosin (HE) staining, we found that the structures of the glomeruli and renal tubules of the cortex and medulla were maintained in the cKO kidney and no obvious embryological phenotype was observed (Fig. S2). Similarly, in adult mice aged 6–8 weeks, the kidneys were harvested and kidney morphology was assessed in detail. On HE staining and assessment of various renal tubule markers, no obvious differences were observed between control and cKO mice (Figs S3 and S4). Additionally, on blood biochemistry assessments and urinalysis, no differences were observed between control and cKO mice (Supplementary Table S1). These results suggest that in the steady state, cKO does not affect the morphology and function of the kidney.

### Fibrosis after IRI was mild in Gcm1 cKO mice

**Figure 3:**
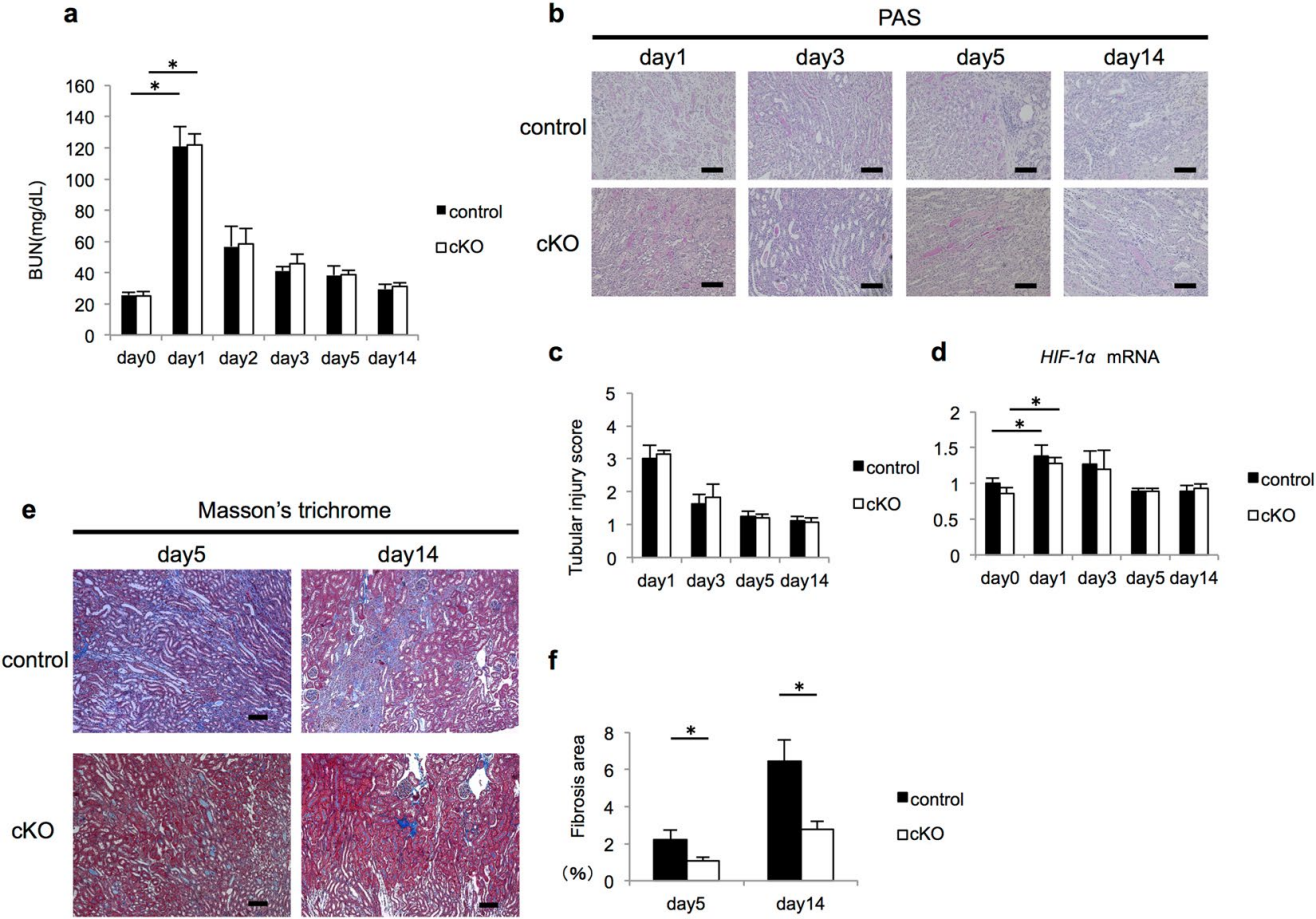
Knockout of *Gcm1* attenuates kidney fibrosis after ischemia–reperfusion injury (IRI). (**a**) Blood urea nitrogen (BUN) level is measured as an indicator of renal function in control and *Gcm1* conditional knockout (cKO) mice before IRI (day 0) or after IRI (ischemia followed by 1, 2, 3, 5, and 14 days of reperfusion). Data are presented as mean ± SEM (*n* = 9). *P < 0.01. (**b**) Representative morphology on periodic acid–Schiff staining (PAS) of kidney sections from control (upper column) and cKO (lower column) mice after IRI (ischemia followed by 1, 3, 5, and 14 days of reperfusion). Scale bars = 100 μm. (**c**) Semiquantitative assessment of tubular injury in control and cKO mice on days 1, 3, 5, and 14 after IRI. Data are presented as mean ± SEM of evaluations in each group (*n* = 5 per group). (**d**) *HIF-1α* expression assessed using real-time polymerase chain reaction in the kidneys of control and cKO mice before IRI (day 0) or after IRI (ischemia followed by 1, 3, 5, and 14 days of reperfusion). Data are presented as mean ± SEM (*n* = 6). *P < 0.05. (**e**,**f**) Analysis of fibrosis after acute kidney injury following IRI. (**e**) Representative morphology on Masson’s trichrome staining of kidney sections from control (upper column) and cKO (lower column) mice after IRI (ischemia followed by 5 and 14 days of reperfusion). Scale bars = 100 μm. (**f**) Semiquantitative assessment of fibrosis on Masson’s trichrome staining in control and cKO mice on days 5 and 14 after IRI. The area of positive staining (green for trichrome staining) is summarized. Data are presented as mean ± SEM of evaluations in each group (*n* = 5 per group). *P < 0.05.

As mRNA levels of *Gcm1*, which is expressed in the cortex region and corticomedullary junction, changed after IRI, function analysis of *Gcm1* after IRI was performed using cKO mice. First, through biochemical assessment of blood, we examined whether the degree of renal dysfunction caused by IRI differed between control and cKO mice. In both groups of mice, the peak BUN level was noted on day 1 after IRI, and no significant differences in renal dysfunction were observed on biochemical assessment (Fig. 3a). Next, we evaluated the morphological differences between control and cKO mice after IRI by periodic acid–Schiff (PAS) staining (Fig. 3b). The tubular injury score after IRI, which was evaluated by PAS staining to quantify the differences in injury between control and cKO mice, did not differ between the two (Fig. 3c). It has been reported that *HIF-1α* is activated for organ protection against hypoxia and ischemic injury^36^. However, the stable expression of *HIF-1α* leads to the progression of fibrosis, causing organ damage^37^. We evaluated whether there was a difference in *HIF-1α* expression after IRI between control and cKO mice. *HIF-1α* expression showed a similar trend after IRI in control and cKO mice (Fig. 3d), indicating that the degree of ischemic injury was not different between control and cKO mice.

We also assessed the degree of fibrosis after IRI with Masson’s trichrome staining (Fig. 3e) and Sirius red staining (Fig. S5a). After the recovery phase of IRI (days 5 and 14), Masson’s trichrome staining showed that the fibrosis area (staining region of collagen fibers) was smaller in cKO mice than in control mice (Fig. 3f). Additionally, on Sirius red staining, the fibrosis area (ratio of the Sirius red-positive area [collagen I (ColI), III]) was significantly smaller in cKO mice than in control mice (Fig. S5b), similar to the finding with Masson’s trichrome staining (Fig. 3f). These results suggested that the degree of fibrosis was lower in cKO mice than in control mice.

### Expression of fibrosis-related genes after IRI decreased in Gcm1 cKO mice

**Figure 4:**
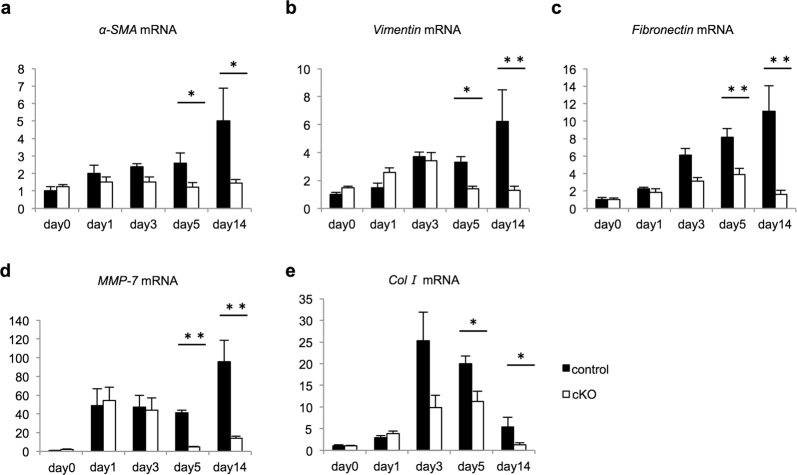
Knockout of *Gcm1* decreases the mRNA expression of various fibrosis-related genes after ischemia–reperfusion injury (IRI). (**a–e**) Expression of fibrosis-related genes in the kidneys from control and *Gcm1* conditional knockout (cKO) mice before IRI (day 0) or after IRI (ischemia followed by 1, 3, 5, and 14 days of reperfusion). *α-SMA* (**a**), *vimentin* (**b**), *fibronectin* (**c**), *MMP-7* (**d**), and *collagen I* (*col I*) (**e**) are assessed. Data are presented as mean ± SEM (*n* = 6). *P < 0.05 vs. control; **P < 0.01 vs. control.

**Figure 5:**
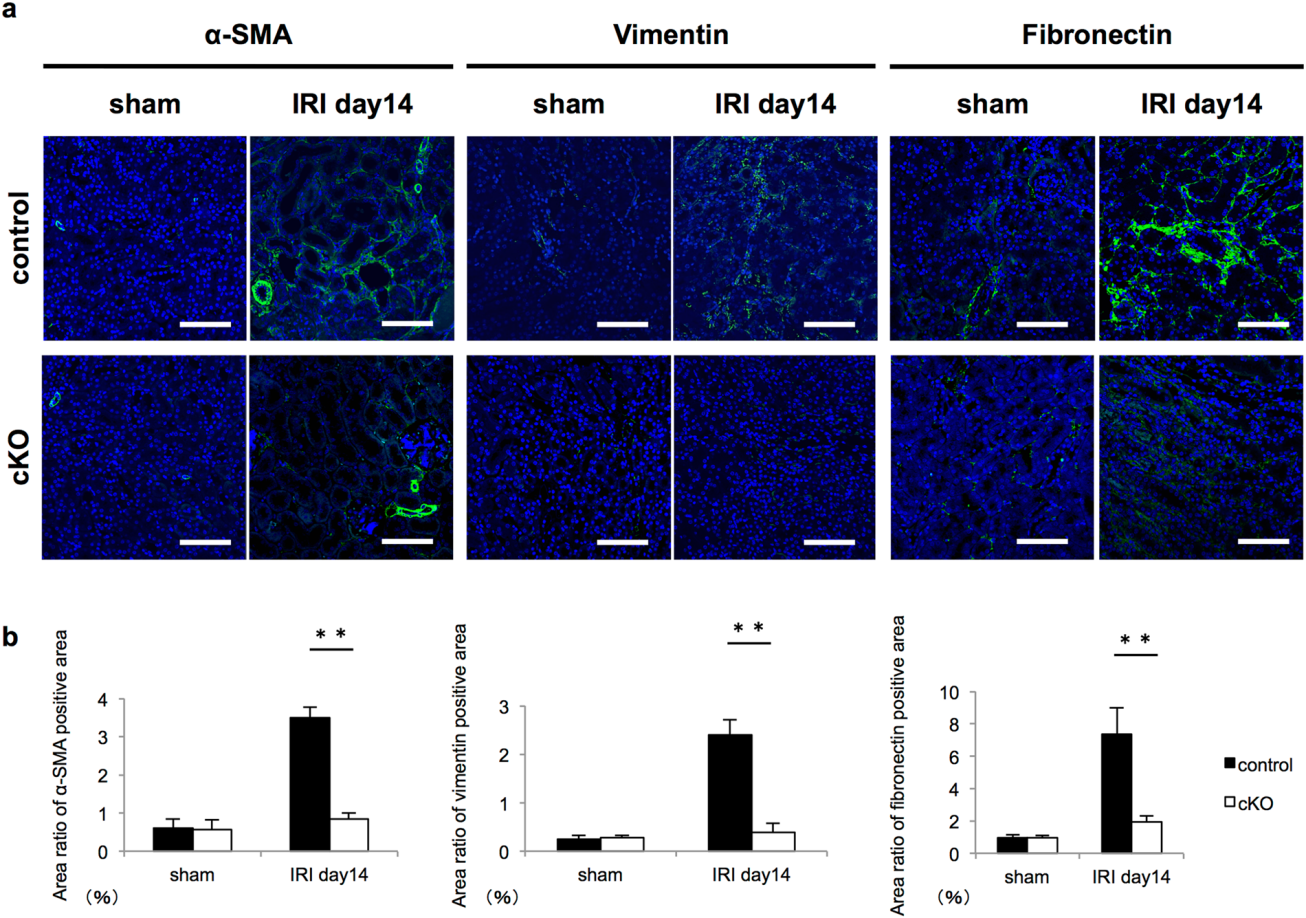
Knockout of *Gcm1* decreases the protein expression of various fibrosis-related genes after ischemia–reperfusion injury (IRI). (**a**) Immunofluorescence staining for α-SMA (left panels), vimentin (middle panels), and fibronectin (right panels) with DAPI. The findings in control mice are presented in the upper panels, and the findings in *Gcm1* conditional knockout (cKO) mice are presented in the lower panels. Protein expression of various fibrosis-related genes is shown in green. DAPI staining of cell nuclei is shown in blue. Scale bars = 100 μm. (**b**) Bar graphs showing quantitative findings of α-SMA, vimentin, and fibronectin on immunostaining of kidney sections from control and cKO mice on day 14 after the sham operation or IRI. **P < 0.01 vs. controls (*n* = 4).

The mechanism of fibrosis in the kidney involves the activation and proliferation of fibroblasts (myofibroblasts), resulting in excessive extracellular matrix production^21^. Therefore, we evaluated the expression of myofibroblast, fibroblast, extracellular matrix, and fibrosis marker genes using real-time qRT-PCR and immunostaining. After the recovery phase of IRI (days 5 and 14), the expressions of the myofibroblast marker alpha-smooth muscle actin (*α-SMA*) (Fig. 4a), fibroblast marker *vimentin* (Fig. 4b), extracellular matrix marker *fibronectin* (Fig. 4c) and fibrosis markers *MMP-7* (Fig. 4d), and *ColI* (Fig. 4e) were significantly lower in cKO mice than in control mice. Additionally, on immunostaining, α-SMA, vimentin, and fibronectin expressions were significantly lower in cKO mice than in control mice (Fig. 5a,b). These results indicated that fibrosis was lower in cKO mice than in control mice.

### Cell proliferation decreased after IRI in Gcm1 cKO mice

**Figure 6:**
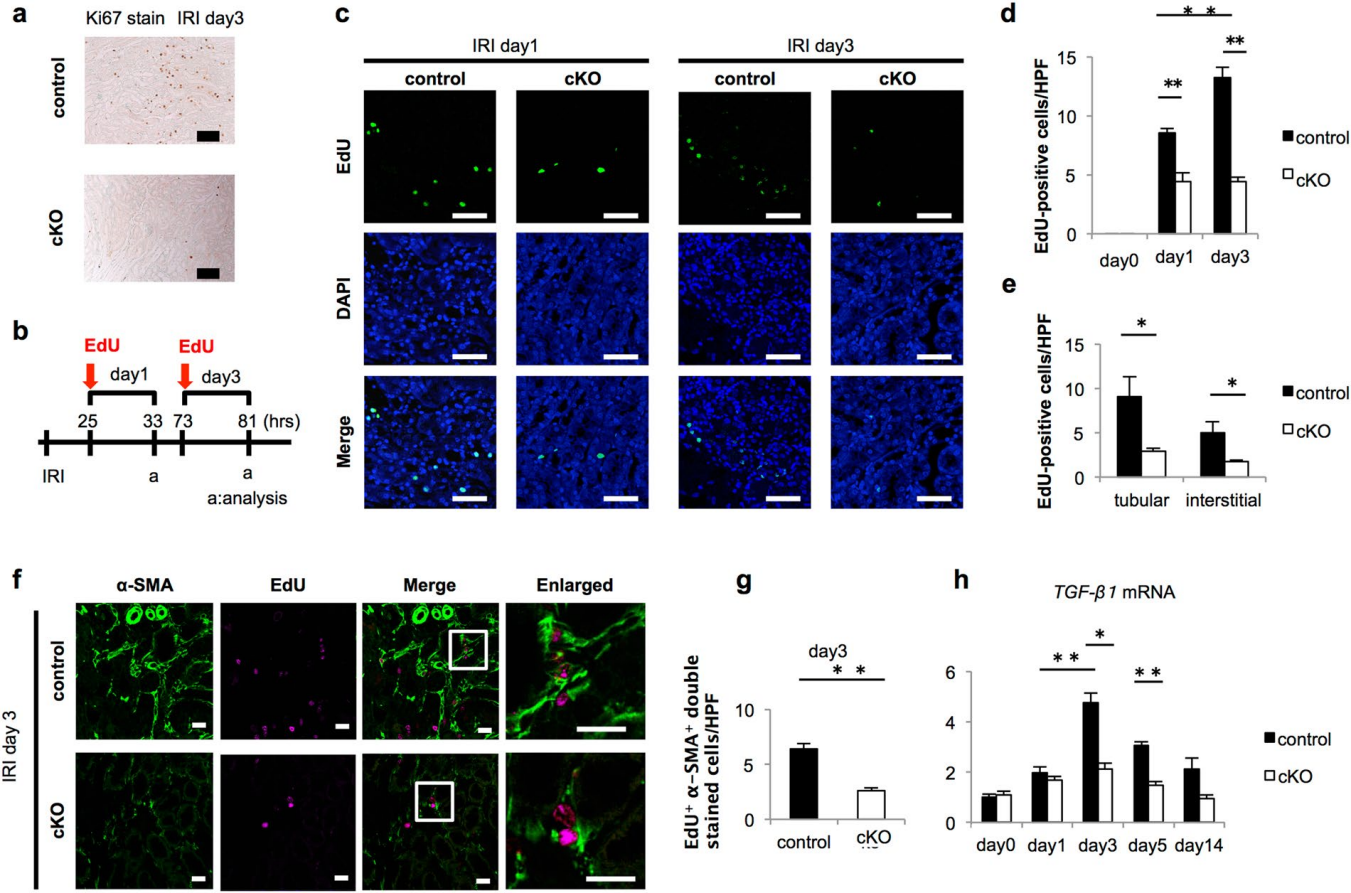
Knockout of *Gcm1* reduces myofibroblast proliferation after ischemia–reperfusion injury (IRI). (**a**) Immunohistochemical analysis of Ki67 staining in the kidney on day 3 after IRI. The findings in control mice are presented in the upper panels, and the findings in *Gcm1* conditional knockout (cKO) mice are presented in the lower panels. Scale bars = 100 μm. (**b**) Schema of the experimental design of 5-ethynyl-2′-deoxyuridine (EdU) injection. Some mice received intraperitoneal injections of EdU 8 h before kidney harvest. (**c**) EdU staining (green) on days 1 and 3 after IRI in control and cKO mice. DAPI staining of cell nuclei is shown in blue. Scale bars = 50 μm. (**d**) Quantitative assessment of EdU-positive cells per high-power field (HPF) in control and cKO mice. Data are presented as mean ± SEM for five mice in each group. **P < 0.01 control vs. cKO. (**e**) Quantitative assessment of EdU-positive tubular and interstitial cells per HPF in control and cKO mice. Data are presented as mean ± SEM for five mice in each group. *P < 0.05 control vs. cKO. (**f**) Double immunofluorescence staining shows co-staining of EdU (magenta) and α-SMA (green) in the kidneys of control and cKO mice on day 3 after IRI. α-SMA is used as a type-specific marker of myofibroblasts. Boxed areas are enlarged in the right panels. Scale bars = 50 μm. (**g**) Quantitative assessment of EdU and α-SMA-positive cells per HPF in control and cKO mice. Data are presented as mean ± SEM for five mice in each group. **P < 0.01 control vs. cKO. (**h**) *TGF-β1* expression assessed using real-time polymerase chain reaction in the kidneys of control and cKO mice before IRI (day 0) or after IRI (ischemia followed by 1, 3, 5, and 14 days of reperfusion). *P < 0.05; **P < 0.01, *n* > 4 at each time point.

In the kidney after IRI, renal tubular regeneration actively occurs together with fibrosis, and cell proliferation actively occurs in the regeneration process^38^. On day 3 after IRI, *Gcm1* expression increased. We determined whether the cell proliferative capacity differs between control and cKO mice with Ki67 staining. In control mice, the number of Ki67-positive cells increased after IRI. However, in cKO mice, the number of Ki67-positive cells clearly decreased (Fig. 6a), and it was suggested that cell proliferation was especially decreased in the recovery phase of IRI. To investigate the cause for this difference, we assessed cell death in the corticomedullary junction of the kidney after IRI in control and cKO mice. The numbers of TUNEL-positive cells in both control and cKO mice were counted, and there were no significant differences in the numbers on days 1 and 3 after IRI. In both groups of mice, the number of TUNEL-positive cells was the highest on day 1 when renal function was the most reduced, and the number of TUNEL-positive cells on day 3 tended to decrease to about one-third the number on day 1 (Fig. S6a,b). These results suggested that the degree of cell death owing to IRI was not different between control and cKO mice and that there was a difference only in cell proliferation. To analyze cell proliferation after IRI in more detail, we assessed the number of cells in which 5-ethynyl-2′-deoxyuridine (EdU) was incorporated on days 1 and 3 after IRI (when cell proliferation occurred). EdU was injected intraperitoneally 25 and 73 h after IRI, and 8 h after each injection of EdU, the kidneys were harvested and observed (Fig. 6b). The number of EdU-positive cells in cKO mice was about half the number in control mice on day 1, and the cell proliferative capacity in cKO mice decreased on day 1 after IRI. Similarly, the number of EdU-positive cells in cKO mice was about one-third the number in control mice on day 3, and the cell proliferative capacity in cKO mice decreased even on day 3 after IRI (Fig. 6c,d). In addition, we analyzed whether the proliferating cells were tubular or interstitial cells on day 3, which is the point when cell proliferation is considered the most increased after IRI^39,40^. When the number of EdU-positive cells was evaluated by dividing the cells into tubular and interstitial cells, the proliferative capacity with regard to both tubular and interstitial cells was significantly lower in cKO mice than in control mice, and the difference was especially prominent for renal tubular cells (Fig. 6e and Fig. S6c). To elucidate whether *Gcm1* has some function in cell proliferation, we overexpressed *Gcm1* in HEK293 and examined the cell proliferation ability with EdU. *Gcm1* transfected cells had more EdU positive cells compared with control cells. (Fig. S7). These results indicated that *Gcm1* overexpression promotes cell proliferation.

To confirm whether the proliferating cells in the interstitium after IRI are myofibroblasts, α-SMA immunostaining and EdU staining were performed on day 3 after IRI (Fig. 6f). There were many α-SMA and EdU double-positive cells in control mice (Fig. 6f). The number of double-positive cells was significantly lower in cKO mice than in control mice (Fig. 6g). These results indicated that myofibroblast proliferation in cKO mice was reduced.

Transforming growth factor-β1 (TGF-β1) induces fibroblast proliferation and activation after IRI^22^; thus, we evaluated *TGF*-*β1* expression (Fig. 6h). In control mice on day 3 after IRI (remarkable difference was noted in *Gcm1* expression [Fig. 1b]), *TGF-β1* expression increased (Fig. 6h). However, in cKO mice, no significant increase in *TGF-β1* expression was noted (Fig. 6h). This result suggested that *Gcm1* in the kidney promoted tubular and interstitial cell proliferation after IRI. With a focus on the interstitium, it was shown that *Gcm1* was related to the regulation of myofibroblast proliferation associated with fibrosis after ischemic injury, and it was suggested that a reduction in cell proliferation is associated with less fibrosis after IRI.

### Gcm1 and TGF-β1 are co-expressed in the proximal renal tubule at the corticomedullary junction after ischemia–reperfusion injury

**Figure 7:**
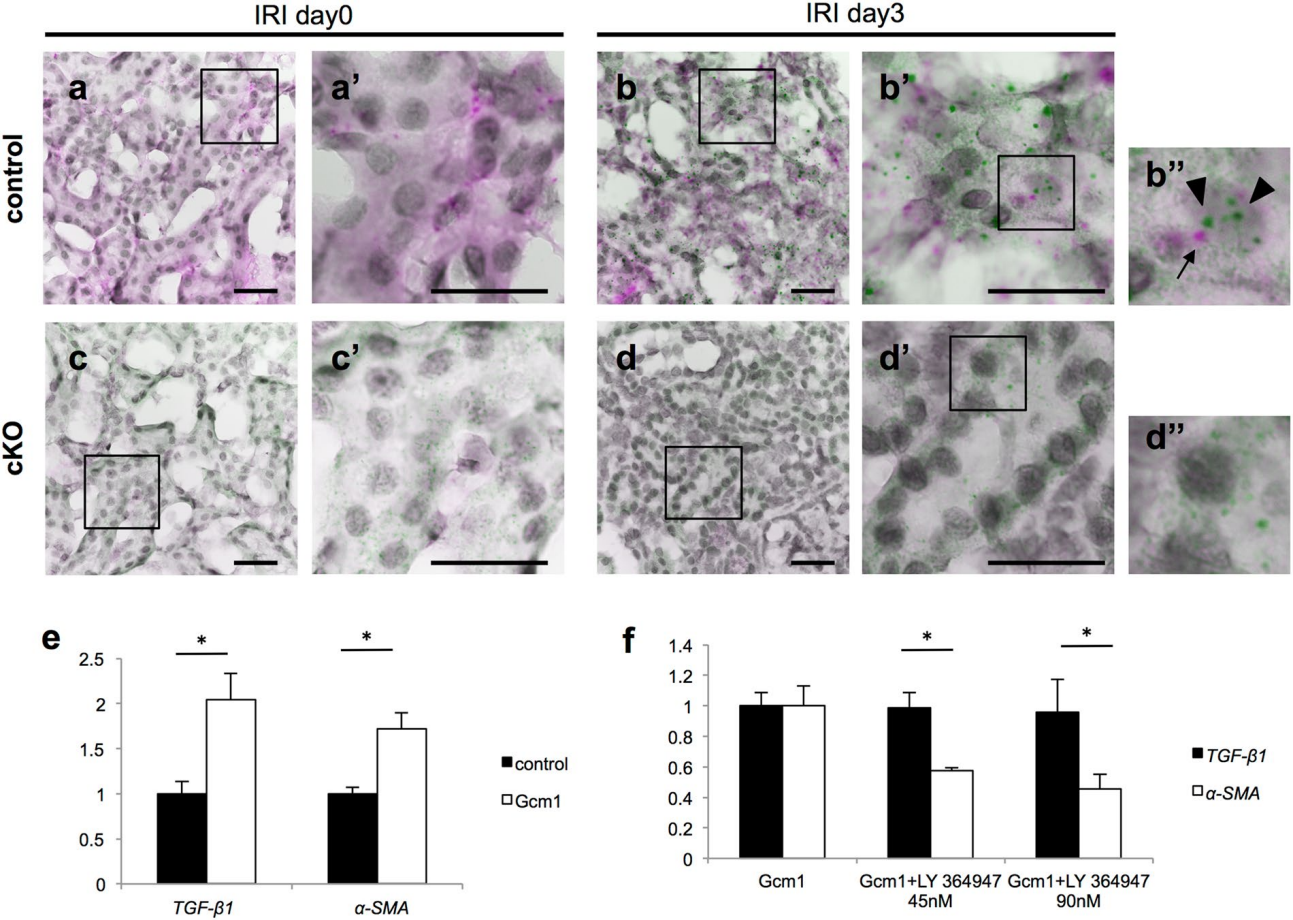
*Gcm1* and *TGF-β1* are co-expressed in the proximal renal tubule after ischemia–reperfusion injury (IRI) and *in vitro* assay. (**a–d**) Representative morphology on ultrasensitive RNA *in situ* hybridization of kidney sections from control (**a**,**b**: upper column) and cKO (**c**,**d**: lower column) mice on day 0 and day 3 after IRI. Boxed areas are enlarged in the right panels (**a′**, **b′**, **b″**, **c′**, **d′** and **d″**). *TGF- β1* is shown in green. *Gcm1* is shown in magenta. Hematoxylin staining of cell nuclei is shown in gray. Arrows indicate *Gcm1* signals, and arrowheads indicate *TGF-β1* signals. Scale bars = 200 μm. (**e**) *TGF-β1* and *α-SMA* expression assessed using real-time polymerase chain reaction in cells transfected with empty vector plasmid DNA (control) and vector plasmid DNA containing *Gcm1* (Gcm1). Data are presented as mean ± SEM (*n* = 6). *P < 0.05. (**f**) Effect of a TGFBR1-specific inhibitor (LY-364947) on *TGF-β1* and *α-SMA* expression assessed using real-time polymerase chain reaction in cells transfected with vector plasmid DNA containing *Gcm1* (Gcm1) and cells transfected with vector plasmid DNA containing *Gcm1* with the inhibitor (Gcm1+ LY-364947 45 nM and 90 nM). Data are presented as mean ± SEM (*n* = 4). *P < 0.05.

To elucidate the role of *Gcm1* in kidney injury and repair associated with IRI, it is important to know the precise localization of *Gcm1* after IRI. Normally, *TGF-β1* expression increases after IRI, but it was found to decrease in cKO mice (Fig. 6h). This result suggests that *Gcm1* is involved in the expression of *TGF-β1* after IRI. To investigate whether *Gcm1* is directly or indirectly involved in the expression of *TGF-β1*, it is necessary to know the details of cells expressing *Gcm1* and *TGF-β1*. We confirmed their expression by *in situ* hybridization, but we could not confirm the expression of *TGF-β1*. Therefore, we used ultrasensitive RNA *in situ* hybridization with RNAscope. We had examined day 0 kidney and 3 days after IRI kidney, which indicated the highest expression of *Gcm1* after IRI, in control and compared with cKO mice kidney. In control mice, expression of *Gcm1* was found in the renal tubules, especially in the corticomedullary junction (Fig. 7a, a′ and S8). *Gcm1* expression was increased after IRI (Fig. 7b, b′ and b″). *TGF-β1* expression was scarcely observed in day 0 kidney (Fig. 7a, a′) but was remarkably observed after IRI, and many cells co-localized with *Gcm1* were observed in the corticomedullary junction (Fig. 7 b″). *TGF-β1* expressing cells not co-localized with *Gcm1* also existed. In cKO mice, *Gcm1* expression was not observed in either day 0 or after IRI kidneys (Fig. 7c, c′, d, d′ and d″), and *TGF-β1* expression was decreased more in cKO mice than in control mice (Fig. 7d, d′ and d″).

### Gcm1 increases α-SMA expression by upregulating TGF-β1 expression *in vitro*

After IRI, *Gcm1* and *TGF-β1* were expressed in the same cells of the proximal renal tubules at the corticomedullary junction, suggesting the association between *Gcm1* and *TGF- β1*. *In vitro* experiments were then conducted to investigate whether the transient increase in expression of *Gcm1* after IRI directly increases *TGF-β1* expression. *TGF-β1* acts as a common inducer of *α-SMA* expression and collagen and fibronectin synthesis in fibroblasts. *TGF-β1* plays an important role in myofibroblast differentiation during wound healing and fibrocontractive diseases by regulating the expression of *α-SMA* in myofibroblasts^41,42^. To also investigate the effect of *Gcm1* overexpression on *α-SMA* leading to fibrogenesis, we evaluated the expression of *TGF-β1* and *α-SMA* using real-time qRT-PCR in cells transfected with empty vector plasmid DNA (control) and vector plasmid DNA containing the *Gcm1* gene. We found that the expression of *TGF-β1* and *α-SMA* was higher in the group overexpressing the *Gcm1* gene than in the control group (Fig. 7e). Next, to confirm whether *Gcm1* increases the expression of *α-SMA* via *TGF-β1*, we conducted an experiment treating cells overexpressing *Gcm1* with a TGFβR1-specific inhibitor (LY-364947) that inhibits TGF-β signaling pathways. We evaluated the expression of *TGF-β1* and *α-SMA* using real-time qRT-PCR in the group overexpressing *Gcm1* and the group overexpressing *Gcm1* treated with LY-364947. In cultured cells supplemented with LY-364947, the expression of *TGF-β1* was similar to that in untreated cultures, but the expression of *α-SMA* was suppressed (Fig. 7f). These results indicate that *Gcm1* promotes the expression of *TGF-β1*, thereby increasing the expression of *α-SMA*.

### Analysis of Gcm1-related genes after acute kidney injury following IRI

To further investigate whether there is a *Gcm1*-related gene other than *TGF-β1* which directly leads to fibrosis, we evaluated the expression of genes, such as the Wnt family and FZD5, which are associated with *Gcm1* as reported in a placenta study^43,44^. In the kidney, sustained activation of Wnt signaling reportedly drives the progression of AKI to CKD^45^. However, among these genes, there was no clear change in *Gcm1* cKO mice after IRI in the kidney (Figs. S9a,b). Therefore, other than *TGF-β1*, we could not find any *Gcm1*-related gene leading to fibrosis.

## Discussion

Our findings indicated that *Gcm1* expression changes during ischemic injury and that *Gcm1* is involved in fibrosis and cell proliferation after kidney tissue injury.

*Gcm1* complete KO is embryonically lethal at E10.5^32^, because *Gcm1* is essential for placental formation. Thus, in previous studies, the function of *Gcm1* was unknown in organs, except for the placenta. We analyzed its function in the kidney using cKO mice (*Gcm1* was knocked out in the kidney). In the kidney, *Gcm1* is known to be expressed in the S3 segment; however, our analysis revealed that it is widely expressed in the renal tubules at the cortex region and corticomedullary junction. We knocked out *Gcm1* in the entire nephron, which is derived from the metanephric mesenchyme, by crossing Wt1^*GFPCre*/+^ mice with *Gcm1*-floxed mice. This approach made it possible to completely knock out *Gcm1* in the kidney. Interestingly, *Gcm1* is also expressed in the developing kidney^29^; however, we could not identify any difference between control and cKO mice. In addition, even after birth, there were no differences in electrolytes on blood biochemical tests and urine biochemical tests between control and cKO mice. These results indicate that *Gcm1* expressed in the renal tubules is not an essential gene for kidney differentiation and development and does not have a biochemical effect in the steady state.

We evaluated renal function by assessing the BUN level and the results showed that renal function did not differ between control and cKO mice in the recovery phase of IRI. However, changes in cell proliferation and fibrosis were observed between control and cKO mice kidney after IRI. With regard to fibrosis, it has already been reported that the production of extracellular matrix begins to rise around 3 days after IRI^46^, and we found that the expression of *Gcm1* transiently increased at the same time. In addition, it was shown that the expression of fibrosis-related genes decreased when *Gcm1* was knocked out. These findings suggest that *Gcm1* has a function related to fibrosis after IRI. Furthermore, some reports have shown that the expression of several genes in the S3 segment increases after IRI, and these genes are involved in kidney fibrosis formation via various mechanisms associated with angiogenesis and the inflammatory response^27,28^. There are several molecules related to fibrosis, and we need to investigate the relationship between them and *Gcm1* in the future.

Normally, with severe ischemia injury, cell death (renal tubule necrosis and apoptosis) occurs, and viable tubular cells undergo cell division and replace the lost cells^47^. It has been shown that the replacement cells ultimately form normal epithelial cells^48^. Some reports have mentioned that peak cell proliferative capacity is noted 3–4 days after IRI^39,40^. Consistent with the results of previous reports, our results indicate that cell proliferation in control mice increased on day 3 rather than on day 1 after IRI. However, in cKO mice, increasing cell proliferative capacity could not be confirmed on day 3 after IRI. Thus, compared with control mice, the usual cell proliferation response to IRI did not occur in cKO mice. Previous studies have reported that when the proliferative capacity of renal tubular cells decreases after IRI, kidney repair remains incomplete and adequate recovery of renal function does not occur^49–51^. However, there was no difference between cKO and control mice in the degree of kidney injury, even though there was a difference in cell proliferation. In the cell proliferation experiments, a small number of proliferating cells was observed in cKO mice, and the cells were probably involved in kidney regeneration. Analysis of long-term observations after IRI will evaluate whether there is more difference in kidney regeneration between control and cKO mice. *Gcm1* gene is one of the mammalian homologs of the *Gcm* gene essential for glial cell development in Drosophila and *Gcm2*, other *Gcm* family, is essential for parathyroid development^52^. There are several reports on the relationship between *Gcm* and cell proliferation. In Drosophila, *Gcm* overexpression was found to promote glial cell proliferation^53^, and knockout of *Gcm2* expression in mice was found to be lead to reduce parathyroid cell proliferation^54^. Therefore, the *Gcm1* gene may also be involved in cell proliferation. In our study, *Gcm1* overexpression was found to promote cell proliferation *in vitro* (Fig. S7), and *Gcm1* knockout was found to inhibit cell proliferation after ischemic kidney injury *in vivo* (Fig. 6c,d). These results suggest that *Gcm1* might be involved in cell proliferation.

Further, what is affected by the reduced cell proliferation by *Gcm1* KO in mice kidney? Another remarkable difference between control and cKO mice after IRI is a decrease in fibrosis. Tissue fibrosis, revealed by histology and qRT-PCR, was much more prominent in control mice than in cKO mice. In general, injured tubules are associated with cell proliferation, and at injury, growth factors are produced from renal tubular epithelial cells and proliferation of fibroblasts occurs^42^. Previous reports have mentioned that the main growth factor is *TGF-β1*, which reportedly activates myofibroblasts and promotes fibrosis after IRI^22,55,56^. A previous study involving IRI also reported that the number of α-SMA and EdU double-positive cells decreased and fibrosis reduced through interference of the production of fibroblast growth factor by renal tubules^21^. Our results indicate that in cKO mice after IRI, there was a clear decrease in fibroblast proliferative capacity in the interstitium, and the expression of *TGF-β1* decreased. These results suggest that the factors associated with the proliferation of fibroblasts may be downstream of *Gcm1*. Furthermore, by ultrasensitive RNA *in situ* hybridization, we found that *Gcm1*-expressing cells also expressed *TGF-β1* and we showed by *in vitro* experiments that *Gcm1* increased *α-SMA* via *TGF-β1*. Our results may demonstrate an association between *Gcm1* and *TGF-β1* in kidney fibrosis. Since expression of *TGF-β1* is observed even in cKO mice (Fig. 7c,d), *TGF-β1* can be induced by IRI even in the absence of *Gcm1*. However, fibrosis in cKO mice was considered to be mild, since the expression of *TGF-β1* was significantly decreased as compared with control mice. A previous study reported that the cell proliferative capacity of tubular and interstitial cells decreased and fibrosis was mild after knocking out a gene related to the cell cycle or growth factor in mice and that the change in cell proliferation was related to fibrosis^57,58^. Our findings suggest that KO of *Gcm1* reduces the cell proliferative capacity of renal tubular cells, then decreases the expression of factors related to the proliferation of fibroblasts produced from renal tubular cells, decreases the interstitial cell proliferation capacity, and indirectly makes fibrosis mild. Moreover, KO of *Gcm1* did not affect kidney tissue damage evaluated by tubular injury score. These results suggest that KO of *Gcm1* is involved in fibrosis rather than in repair of tissue after AKI. Therefore, we speculate that there might be two independent functional cells associated with repair and fibrosis occurring after IRI. *Gcm1* is involved in cell proliferation associated with this fibrosis, and it may not be directly involved in the proliferation of cells for repair. However, since renal dysfunction due to fibrosis is not reflected early in serologic renal function, it is presumed that the recovery of renal function, as assessed by BUN level, did not significantly differ between control and cKO mice in the present study. In the future, detailed analysis of the fibrosis caused to various degrees of kidney damage is desired.

In addition, in this study, we showed that *Gcm1* expression transiently decreased immediately on days 1 and 2 after IRI, and the expression sharply increased on day 3 after IRI, which is the recovery phase of renal function. However, we could not clarify the reason for the change. Some studies have shown that *Gcm1* is associated with ischemia^59,60^, and it is presumed that even in the kidney, *Gcm1* expression can increase in response to hypoxia by the change in *HIF-1α* after IRI, as shown in the study (Fig. 3d).

To investigate the possibility that the function of GCM1 in mouse is similar to that in the human kidney, we used human transcriptome data from a previous report^61^ and found that *GCM1* expression was observed in the renal tubules (data not shown). In addition, we overexpressed *Gcm1* in HEK 293, which is a human cell and found increased expression of *TGF-β1* and *α-SMA*. This suggested the possibility that the *Gcm1*-related function associated with fibrosis in mouse kidney exists in human kidney. Therefore, it may be possible to approach new drug discoveries related to the treatment of fibrosis after AKI by regulation of *Gcm1*.

We acknowledge that there are several limitations in this study. First, currently, we only found *TGF-β1* as a factor related to *Gcm1*, which causes myofibroblast proliferation in the interstitium. Among the genes that were reported to be associated with *Gcm1* in a placenta study^43,44^, no clear change was observed in *Gcm1* cKO mice after IRI in the kidney (Fig. S9a,b). Second, we could not clarify why *Gcm1* transiently changes its expression after IRI. Despite these limitations, this study is valid because the function of *Gcm1* in the kidneys was confirmed for the first time. To prevent the progression of AKI to CKD, it may be necessary to analyze the detailed mechanisms of crosstalk among renal tubular and interstitial cells after ischemic injury through *Gcm1* in the future.

In conclusion, *Gcm1* is involved in the mechanisms of fibrosis and cell proliferation after ischemic injury of the kidney. Further analyses on *Gcm1* in kidney injury are expected to enhance our existing knowledge on improving fibrosis occurring in CKD.

## Methods

### Animals

Mice aged 6–8 weeks were used for experiments. For wild-type mice analyses, male C57BL/6 J mice were purchased from Japan Charles River Laboratories (Kanagawa, Japan). The wild-type mice were used for BUN assay and quantitative analysis of *Gcm1* to confirm normal reactivity under the condition of IRI. Homozygous *Gcm1*-floxed mice (Gcm1^*flox*/*flox*^) (C57BL/6 N background) were generated as follows. The BAC clone RP23-463G12 for the target region was obtained from Open Biosystems (Huntsville, AL, USA). *LoxP* and a *FRT*-flanked neomycin-resistance gene cassette (*FRT*-*PGK*-*gb2*-*neo*-*FRT*) (Gene Bridges, Heidelberg, Germany) were inserted using a BAC Subcloning Kit with Red/ET Recombination (Gene Bridges). Exon 3 of *Gcm1* was flanked by *loxP* sites (Fig. 2a). The first *loxP* was inserted into intron 2 (346 bp upstream of exon 3). The *FRT*-flanked neo cassette and second *loxP* were inserted into intron 3 (1827 bp downstream of exon 3). The targeting vector was constructed by subcloning the *loxP*-flanked exon 3 region of *Gcm1* with homologous arms into the DT-A-pA vector (Riken BRC, Tsukuba, Japan). *Gcm1*-floxed mice were generated and provided by Riken BRC through the National Bio-Resource Project of MEXT, Japan. The mouse chimeras were mated with C57BL/6 N female mice, and F1 Gcm1^*flox*/+^ mice were genotyped using genomic PCR (*Gcm1*-floxed mice genotyping, 5′-GGCATAGATAGCACACACCTTGTCG-3′ and 5′-TGAAAGGGTCTTTTGTCCCCTAAAGG-3′; 75 and 109 bp bands were generated for the wild-type and floxed alleles). Homozygous mice were maintained with brother–sister mating over 20 generations. Mutant mice were mated with FLP mice to remove the neomycin-resistance gene in Gcm1^*flox*/*flox*^ mice. Wt1EGFPCre/+ knock-in mice (Wt1tm1[EGFP/cre]Wtp/J, stock no: #010911) were purchased from Jackson Laboratory (Bar Harbor, ME, USA). By mating *Gcm1*-floxed mice with Wt1EGFPCre/+ heterozygous mice (Wt1^*GFPCre*/+^), cKO mice, in which the *Gcm1* gene was specifically disrupted in the metanephric mesenchyme (genotype: Wt1^*GFPCre*/+^; Gcm1^*flox*/*flox*^), were created. These mice were crossbred with homozygous *Gcm1*-floxed mice (genotype: Gcm1^*flox*/*flox*^) to generate 50% Wt1^*GFPCre*/+^; Gcm1^*flox*/*flox*^ mice (cKO mice) and 50% Gcm1^*flox*/*flox*^ mice (control mice) within the same litter. A routine PCR protocol was adopted for genotyping tail DNA samples using KOD Fx (Toyobo, Osaka, Japan) with the following primer pairs: Wt1^*GFPCre*/+^ mice genotyping, 5′-TCCCGCTTGTCACAACTGAC-3′, 5′-CCCTGTCCGCTACTTTCAGA-3′, and 5′-GAACTTCAGGGTCAGCTTGC-3′, which generated 1578 and 1131 bp fragments for wild-type mice and a 1131 bp fragment for Wt1^*GFPCre*/+^ mice. Genomic PCR and RT-PCR were performed to confirm whether *Gcm1* was properly knocked out in the kidney (Fig. 2b,c). We used primer 1 F (5′-GGCATAGATAGCACACACCTTGTCG-3′) and primer 1 R (5′-CCATGAGCCAATCTCTTTCC-3′) for genomic PCR and primer 2 F (5′-ACGTGAAAACGACTGACTGGT-3′) and primer 2 R (5′-AGGAAGCGCCTTCGCTG-3′) for RT-PCR. For genotyping, we used a 1 Kb Plus DNA Ladder (Invitrogen) and simultaneously ran the samples of control and cKO mice. Control mice and cKO mice were used in all experiments.

All animal experiments were approved by the Animal Care and Experimentation Committee of the Jikei University School of Medicine (No. 2015-096C1) and were performed in accordance with approved guidelines.

### Renal IRI model

Mice were anesthetized with isoflurane (2% for induction and 1.5% for maintenance), and a back incision was made. Both renal arteries were clamped for 30 min. After clamp removal, the kidneys were inspected for recovery of blood flow and signs of internal bleeding, and the incision was closed. Sham-operated mice underwent the same procedures but without occlusion of the renal pedicle. The intrarectal temperature was maintained at 36.5–37.5 °C with a heating pad. Mice were euthanized before IRI (day 0), after the sham operation and after IRI. Blood and kidneys were collected and fixed in 4% PFA at 4 °C overnight for various analyses.

### BUN assay

The extent of kidney damage was assessed by analyzing BUN at different time points simultaneously. For analyzing renal function, blood samples were collected from the heart under anesthesia before IRI (day 0) and after IRI. The BUN level was determined using SPOTCHEM (ARKRAY, Kyoto, Japan), according to the protocols specified by the manufacturer.

### Measurement of urine samples

Mice were individually kept in metabolic cages (Tecniplast, Milan, Italy) for 24 h to collect urine samples and determine 24 h urine production. Collected urine was centrifuged at 3000 rpm for 5 min and stored at −20 °C until measured at Oriental Yeast Co., Ltd. (Tokyo, Japan).

### Real-time PCR

Quantitative analyses of the genes for *α-SMA*, *vimentin*, *fibronectin*, *ColI*, *MMP7*, *TGF-β1*, *HIF-1α*, *Gcm1*, and *GAPDH* were performed using real-time PCR (Rotor-Gene Q/RG-6000; Qiagen, Hilden, Germany). Total RNA was extracted from harvested kidney tissue using the TRIzol RNA isolation system (NucleoSpin^®^ RNA, TaKaRa Bio, Shiga, Japan). First-strand cDNA was synthesized from 1 μg of RNA using a reverse transcriptase kit (High-Capacity RNA-to-cDNA™ Kit, Thermo Fisher Scientific, Waltham, MA, USA). Real-time PCR was performed using the Roter-Gene SYBR Green PCR Kit (Qiagen). The PCR reaction involved 40 cycles, and the conditions were as follows: 95 °C for 5 s and 60 °C for 10 s. The specific primer sequences used are mentioned in Supplementary Table S2. Primers for *Wnt* family were previously described^45^. Expression of the various genes was calculated after normalization with *GAPDH*.

### *In situ* hybridization

Fixed specimens were rinsed several times with cold phosphate-buffered saline (PBS), immersed in 30% sucrose (prepared with PBS) at 4 °C, embedded in Tissue Tek OCT compound (4583D; Sakura, Tokyo, Japan), and frozen. The specimens were then sectioned at a thickness of 10 μm using a cryostat (CM3050S; Leica, Tokyo, Japan). Digoxigenin-labeled antisense RNA probes were synthesized using the DIG RNA Labeling Kit (SP6/T7, Roche Diagnostics, Risch-Rotkreuz, Switzerland) with plasmids containing the *Gcm1*, *NaPiIIa*, *Napsa*, and *NKCC2* genes. *In situ* hybridization was performed as previously described^62^ with some modifications.

### Renal histological analysis

Fixed specimens were paraffin-embedded and sectioned at a thickness of 4 µm, according to the standard procedure. The sections were deparaffinized and rehydrated. Tissues were stained with HE, PAS, Masson’s trichrome, and Picrosirius red (Cosmo Bio Company, Ltd., Tokyo, Japan). They were also subjected to immunofluorescence staining, proliferation assay, and TUNEL assay. For the histological analysis of tubulointerstitial injury (tubular necrosis or damage, loss of brush border, tubular dilatation, tubular atrophy, and tubular casts in the sample) after IRI, the sections were stained with PAS. Ten corticomedullary fields were examined in each section at 200× magnification, and a semiquantitative analysis of tubulointerstitial injury was performed. Tubular injury was scored as follows on PAS by estimating the percentage of tubules that showed epithelial necrosis, loss of brush border, or had necrotic debris or cast^63^: 0, none; 1, ≤10%; 2, 11%–25%; 3, 26%–45%; 4, 46%–75%; and 5, >76%. Fibrosis was assessed in similar tissue sections stained with Masson’s trichrome and Sirius red^64^, and 10 fields were randomly selected from each kidney. Masson’s trichrome staining (standard diagnostic protocol) was performed on days 5 and 14 after IRI to estimate tubulointerstitial lesions. For investigating interstitial collagen deposition, paraffin sections were stained with Sirius red. For quantification, regions of interest were defined, and large vessels and glomeruli were excluded from the analysis. The proportion of the Sirius red-positive area was analyzed automatically using Image J software (National Institutes of Health, Bethesda, MD, USA) as a percentage of the area of interest.

### Immunofluorescence staining

Paraffin sections at a thickness of 4 µm were used for immunofluorescence staining. The primary antibodies were against α-SMA (ab5694; Abcam, Cambridge, UK), vimentin (D21H3, #5741, Cell Signaling, Beverly, MA, USA) and fibronectin (F3647, Sigma-Aldrich, St. Louis, MO, USA). The secondary antibodies were Alexa Fluor^®^ 488-conjugated antibodies (Jackson Immuno-Research Laboratories, West Grove, PA, USA). For nuclei staining, 4,6-diamidino-2-phenylindole (DAPI) was used. All sections were visualized under a confocal microscope (LSM880, Carl Zeiss, Oberkochen, Germany). The interstitial areas of α-SMA, vimentin, and fibronectin on immunostaining were quantified in 10 regions of randomly selected fields using Image J software, and the results were expressed as a percentage of the cortical area stained (large blood vessels were excluded from the analysis for α-SMA staining).

### *In vivo* proliferation assay

Ki67 immunostaining was performed on paraffin-embedded sections treated with HistoVT One (Nacalai Tesque, Kyoto, Japan) at 105 °C for 15 min for antigen retrieval. According to the manufacturer’s instructions, tissue sections were incubated with anti-Ki67 primary antibodies (RM-9106; LabVision, Fremont, CA, USA) overnight at 4 °C. Bound primary antibodies were detected using biotinylated goat anti-rabbit IgG antibodies (Vector Laboratories, Burlingame, CA, USA) and the Vectastain Elite ABC standard kit (Vector Laboratories). Tissue sections were stained using DAB-H_2_O_2_ as a substrate.

To directly measure DNA synthesis, the EdU assay was performed. EdU solution was prepared by dissolving 50 mg of EdU (Invitrogen, Carlsbad, CA, USA) in 50 mL of PBS. Mice were administered intraperitoneal injections of EdU (10 mg/kg body weight) 8 h before kidney harvest. The EdU assay was performed using Click-iT^®^ EdU Imaging Kits (Invitrogen), according to the manufacturer’s recommendations. EdU-positive cells were counted separately in 10 randomly selected non-overlapping renal corticomedullary fields (400× magnification) of tubular or interstitial areas per section in each mouse. The results were expressed as the number of EdU-positive cells per field in the tubular region and in the interstitial region.

### TUNEL assay

The TUNEL assay was performed using the *In Situ* Cell Death Detection Kit and fluorescein (Roche, Mannheim, Germany), according to the manufacturer’s recommendations. The number of TUNEL-positive cells in 10 regions of randomly selected corticomedullary fields was counted under a light microscope.

### Plasmid constructs for cell culture assays

Generation of the *pCAGGS* plasmid containing the *Flag*-tagged *Gcm1* (*pCAGGS-Gcm1-Flag*), mouse coding sequence *of Gcm1* was amplified by PCR from mouse kidney cDNA using the primers: forward, 5′-CATGCCATGGAACTGGACGACTTTGAT-3′ and reverse, 5′-TCTTAAAGAACAGAAGTTTAGGAGCA-3′ to generate an NcoI restriction site in the 5′end. Next, *Flag*-tagged added to *Gcm1* PCR product was performed with the following primers: forward,5′-CATGCCATGGAACTGGACGACTTTGAT-3′ and reverse, 5′-GGAATTCTTACTTGTCATCGTCATCCTTGTAGTCGATGTCATGATCTTTATAATCACCGTCATGGTCTTTGTAGTCTCTTAAAGAACAGAAGT-3′, to generate a *Flag* fragment containing an EcoI restriction site in the 3′end. This fragment was inserted between the NcoI and EcoI restriction sites to generate the *pSlax21-Gcm1-Flag* vector. Subsequently, *Gcm1-flag* sequences were excised from the *pSlax21* vector by ClaI restriction enzyme and inserted into the ClaI site of the *pCAGGS* plasmid to generate *pCAGGS-Gcm1-Flag*. The expression of *pCAGGS-Gcm1*-*Flag* was verified using Flag-antibody (Fig. S10).

### Cell culture assays

HEK293 was cultured in high-glucose Dulbecco’s modified Eagle medium (Wako) with 10% fetal bovine serum (Biowest). Using FuGENE HD transfection reagent (Promega), 1 μg of *pCAGGS* or *pCAGGS-Gcm1-Flag* was introduced into 2.0 × 10^5^ HEK293 cells in 12-well plates according to the protocol. To monitor the proliferation ability, the cells were incubated with 10 μM EdU for 6 h before cell harvest at 24 h and 48 h. After incubation, the cells were washed with PBS and fixed with 4% paraformaldehyde for 15 minutes at room temperature, and then washed again with PBS. The EdU assay was performed using Click-iT^®^ EdU Imaging Kits (Invitrogen) according to the manufacturer’s instructions. Cells were stained with DAPI (Sigma). EdU-positive cells were counted separately in 10 randomly selected non-overlapping fields (400× magnification) in cells transfected with empty vector plasmid DNA and vector plasmid DNA containing *Gcm1*. The results were expressed as the percentage of EdU-positive cells. For inhibition of the TGF-β1 signaling assay, LY-364947 (Sigma) was used at a final concentration of 45 or 90 nM. After 48 h, the cultured cells were washed with PBS twice, and total RNAs were extracted by TRIzol (Thermo Fisher Scientific). Then, 1 μg of total RNA from cells was reacted with DNaseI (Roche) to digest genome DNA. Total RNAs were reverse-transcribed by prime script II (TakaRa) to prepare cDNA for real-time PCR. Real-time PCR was performed using the Roter-Gene SYBR Green PCR Kit (Qiagen). The PCR reaction involved 40 cycles, and the conditions were 95 °C for 5 s and 60 °C for 10 s. The specific primer sequences used are mentioned in Supplementary Table S2. For immunostaining, cells were washed with PBS, fixed with 4% paraformaldehyde in PBS for 10 min at room temperature, and subsequently incubated with the blocking solution for 60 min. Cells were immunostained with a primary antibody (anti-FLAG; F1804, Sigma-Aldrich, St. Louis, MO, USA) in the blocking solution for 1 h at room temperature, and subsequently incubated with the secondary antibody solution for 30 min at room temperature. Cells transfected with empty vector plasmid DNA were used as controls.

### Ultrasensitive RNA *in situ* hybridization assay

Ultrasensitive RNA *in situ* hybridization was performed using fixed frozen tissue sections and the RNAscope 2.5 HD Duplex Detection Kit (ACDBio #322436). We used samples of control and *Gcm1* cKO mice kidney at day 0 and day 3 after IRI. We followed the manufacturer’s protocol. Mm-*Gcm1* (ACDBio #429661) and Mm-*Tgfb1*-C2 (ACDBio #403451-C2) probes were used for the RNAscope assay. We used PhotoShop to identify the color of photos taken with a microscope (Axio Imager D1, Carl Zeiss, Oberkochen, Germany) by copying the red channel and pasting it into the blue channel based on Color Universal Design.

### Statistical analysis

Data are expressed as mean ± standard error of the mean (SEM). Statistical differences were assessed using the Mann–Whitney test. A *P*-value < 0.05 was considered significant.

## Supplementary information

Supplementary Dataset
