# Supplementary material for: Gcm1 is involved in cell proliferation and fibrosis during kidney regeneration after ischemia–reperfusion injury
Source: Sci Rep. 2019 May 27;9:7883. doi: 10.1038/s41598-019-44161-y (PMC6536531; doi:10.1038/s41598-019-44161-y)
Supplement: Supplementary file 1 — Supplementary Dataset [file 41598_2019_44161_MOESM1_ESM.pdf]

Supplementary information

Title

Gcm1 is involved in cell proliferation and fibrosis during kidney regeneration after ischemia-reperfusion injury

Authors

Sahoko Kamejima[1, 2], Norifumi Tatsumi[1], Akane Anraku[1], Hideaki Suzuki[1], Ichiro Ohkido[2], Takashi Yokoo [2], Masataka Okabe[1]

1Department of Anatomy, The Jikei University School of Medicine

2Division of Nephrology and Hypertension, Department of Internal Medicine, The Jikei University School of Medicine

Supplementary Figures S1-S10

Supplementary Tables S1-S2

Supplementary Figure S1: In situ hybridization of *Gcm1* in the kidneys of control and *Gcm1* conditional knockout (cKO) mice

Gcm1 expression is observed in the cortex and corticomedullary junction only in control mice. Scale bars = 500 µm.

Supplementary Figure S2: Representative morphology on hematoxylin–eosin staining of E18.5 kidneys

Representative morphology on hematoxylin–eosin staining of kidney sections from control and *Gcm1* conditional knockout (cKO) mice at E18.5. The left panels are at 25× magnification. The middle panels are at 40× magnification. The right panels (cortex, glomeruli, and tubules) are at 100× magnification.

Supplementary Figure S3: Representative morphology on hematoxylin–eosin staining of adult kidneys

Representative morphology on hematoxylin–eosin staining of kidney sections from control and *Gcm1* conditional knockout (cKO) mice at 6–8 weeks. The left panels are at 25× magnification. The middle panels (corticomedullary junction) are at 100× magnification. The right panels (proximal tubule) are at 400× magnification.

Supplementary Figure S4: Representative morphology revealed by several tubular markers on in situ hybridization of adult kidneys

Representative images showing several tubular markers on in situ hybridization of kidney sections from control and *Gcm1* conditional knockout (cKO) mice at 6–8 weeks. *NaPiIIa* is a marker of the S1 and S2 segments of the proximal tubule; *Napsa* is a marker of the S3 segment of the proximal tubule; and *NKCC2* is a marker of the Henle loop.

Supplementary Figure S5: Analysis of fibrosis after acute kidney injury following ischemia–reperfusion injury (IRI)

(**a**) Representative morphology on Sirius red staining of kidney sections from control (upper column) and *Gcm1* conditional knockout (cKO) (lower column) mice after IRI (ischemia followed by 5 and 14 days of reperfusion). Scale bars = 100 µm.

(**b**) Semiquantitative assessment of fibrosis on Sirius red staining in control and cKO mice on days 5 and 14 after IRI. The area of positive staining (red for Sirius red staining) is summarized. Data are presented as mean ± SEM of evaluations in each group ($n = 5$ per group). *$P < 0.05$.

Supplementary Figure S6: Cell apoptosis and cell proliferation after ischemia–reperfusion injury (IRI) in control and *Gcm1* conditional knockout (cKO) mice

(**a**) TUNEL staining (green) shows apoptotic renal cells in the kidneys on days 1 and 3 after IRI. Arrows indicate TUNEL-positive cells. DAPI staining of cell nuclei is shown in blue. Scale bars = 50 µm.

(**b**) Quantitative assessment of TUNEL-positive cells per high-power field (HPF) in control and cKO mice. Data are presented as mean ± SEM for five mice in each group. **$P < 0.01$.

(**c**) 5-Ethynyl-2′-deoxyuridine (EdU) staining (green) merged with a bright-field image on day 3 after IRI in control and cKO mice. Arrows indicate EdU-positive tubular cells, and arrowheads indicate EdU-positive interstitial cells. DAPI staining of cell nuclei is shown in blue. Scale bars = 50 µm.

Supplementary Figure S7: *Gcm1* overexpression promoted cell proliferation

(**a**) 24 h and 48 h after transfection, EdU cell proliferation assay was performed in cells transfected with empty vector plasmid DNA (control) and vector plasmid DNA containing *Gcm1* (Gcm1). Merge of paired immunofluorescence staining for EdU and

DAPI in each cell line. EdU staining is shown in red. DAPI staining of cell nuclei is shown in blue. Scale bars = 50 μm. (**b**) The percentage of EdU-positive cells in each line was presented for quantitative assessment of EdU-positive cells. Data are presented as mean ± SEM ($n = 6$). *$P < 0.05$.

Supplementary Figure S8: *Gcm1* is expressed in the proximal renal tubule especially corticomedullary junction in kidney.

Representative morphology on ultrasensitive RNA in situ hybridization of kidney sections from control mice on day 0 (upper column) (**a**), (**a**') and day 3 (lower column) (**b**), (**b**') after IRI. Boxed areas are enlarged in the right panels (**a')**, (**b**'). *TGF-β1* is shown in green. *Gcm1* is shown magenta. Hematoxylin and eosin (HE) staining of cell nuclei is shown in gray. Scale bars = 200 μm.

Supplementary Figure S9: Analysis of *Gcm1*-related genes after acute kidney injury following IRI

Expression of *Gcm1*-related genes in the kidneys from control and *Gcm1* cKO mice before IRI (day 0) or after IRI (ischemia followed by 1, 3, 5, and 14 days of reperfusion). Wnt family genes (**a**) and FZD5 (**b**) are assessed. Data are presented as mean ± SEM ($n = 4$). *$P < 0.05$ vs. control.

Supplementary Figure S10: Immunofluorescence staining of Flag-tagged Gcm1 protein transfected into HEK293 cells

The findings in *pCAGGS* (control) are presented in the upper panels, and the findings in *pCAGGS-Gcm1-Flag* are presented in the lower panels. Protein expressions of Flag-tagged is shown in green. DAPI staining of cell nuclei is shown in blue.

Supplementary Table S1: Serum and urine biochemical analyses of basic electrolytes and renal function

Analyses are performed in control and *Gcm1* conditional knockout (cKO) mice at 6–8 weeks. Data are presented as mean ± SEM, n = 10.

Supplementary Table S2: Primers used in the study

Fig. S1

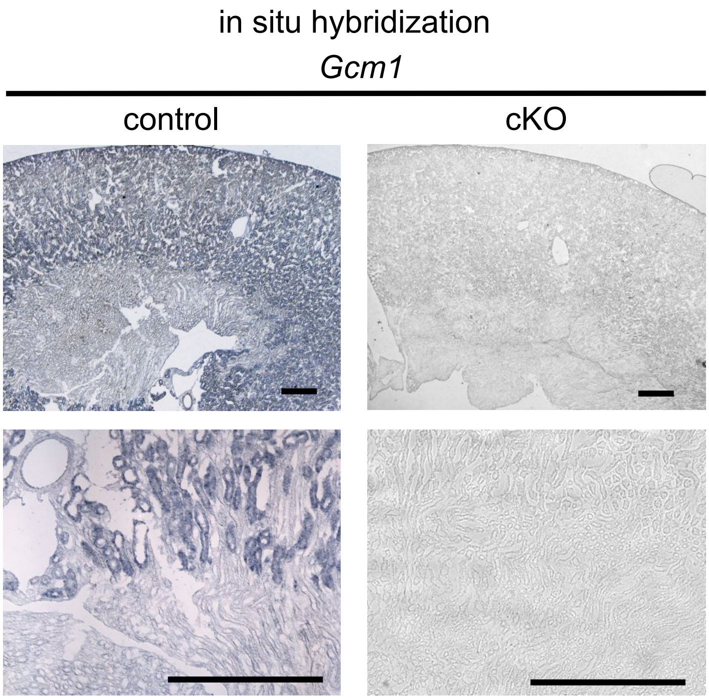

Fig. S2

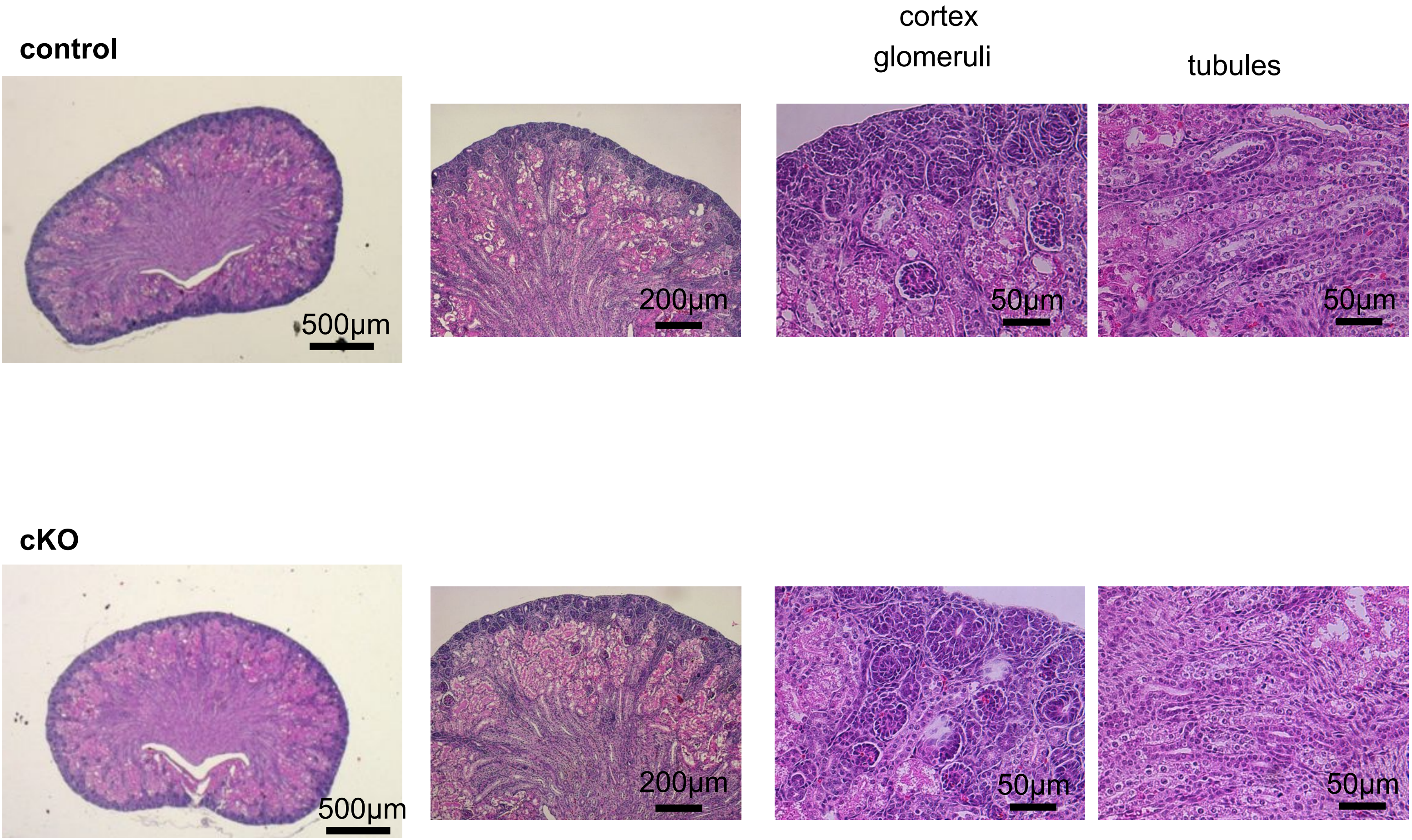
control
cortex
glomeruli
tubules
500μm
200μm
50μm
50μm
cKO
500μm
200μm
50μm
50μm

Fig. S3

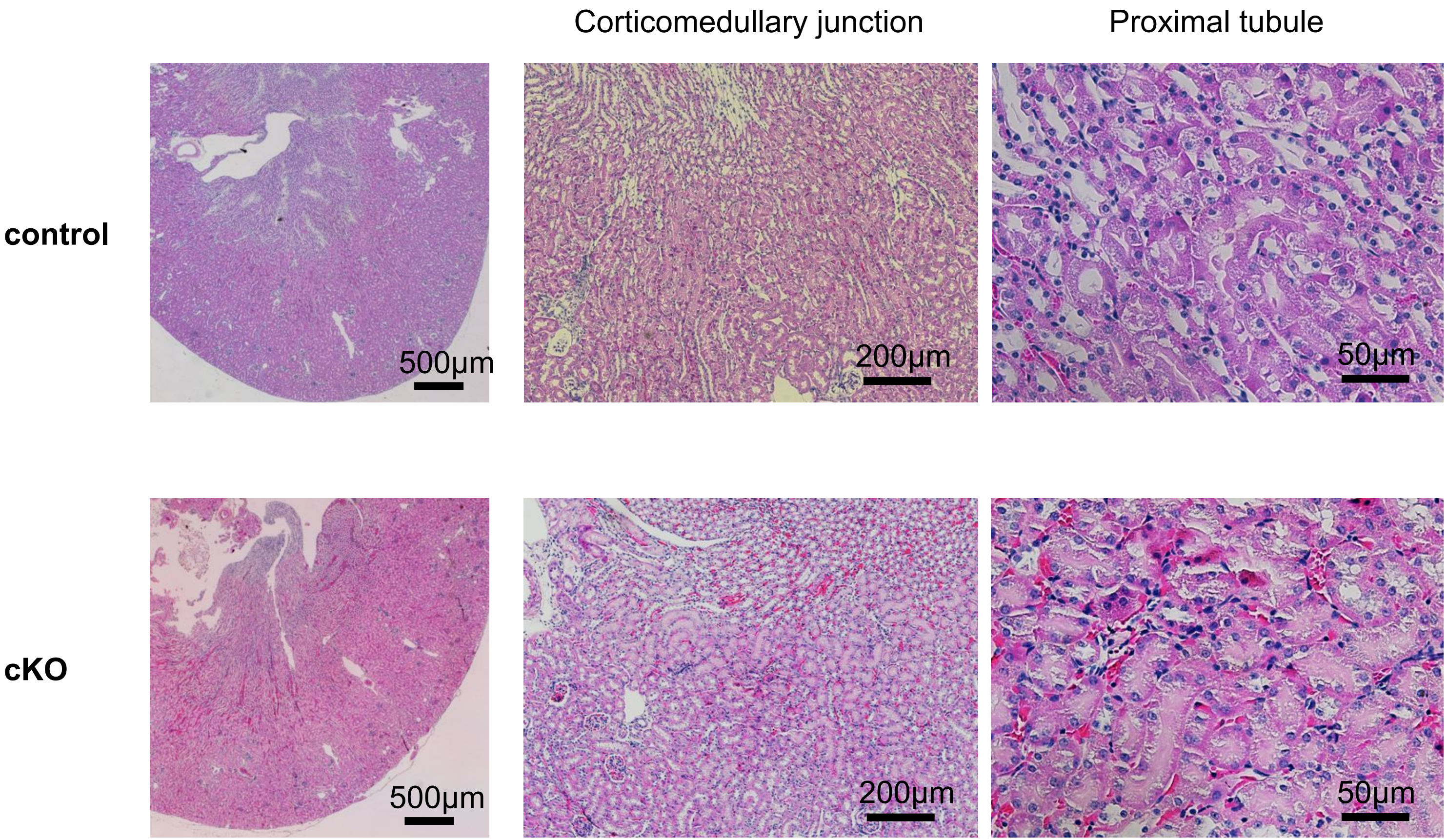

Corticomedullary junction
Proximal tubule
control
cKO
500μm
200μm
50μm
500μm
200μm
50μm

Fig. S4

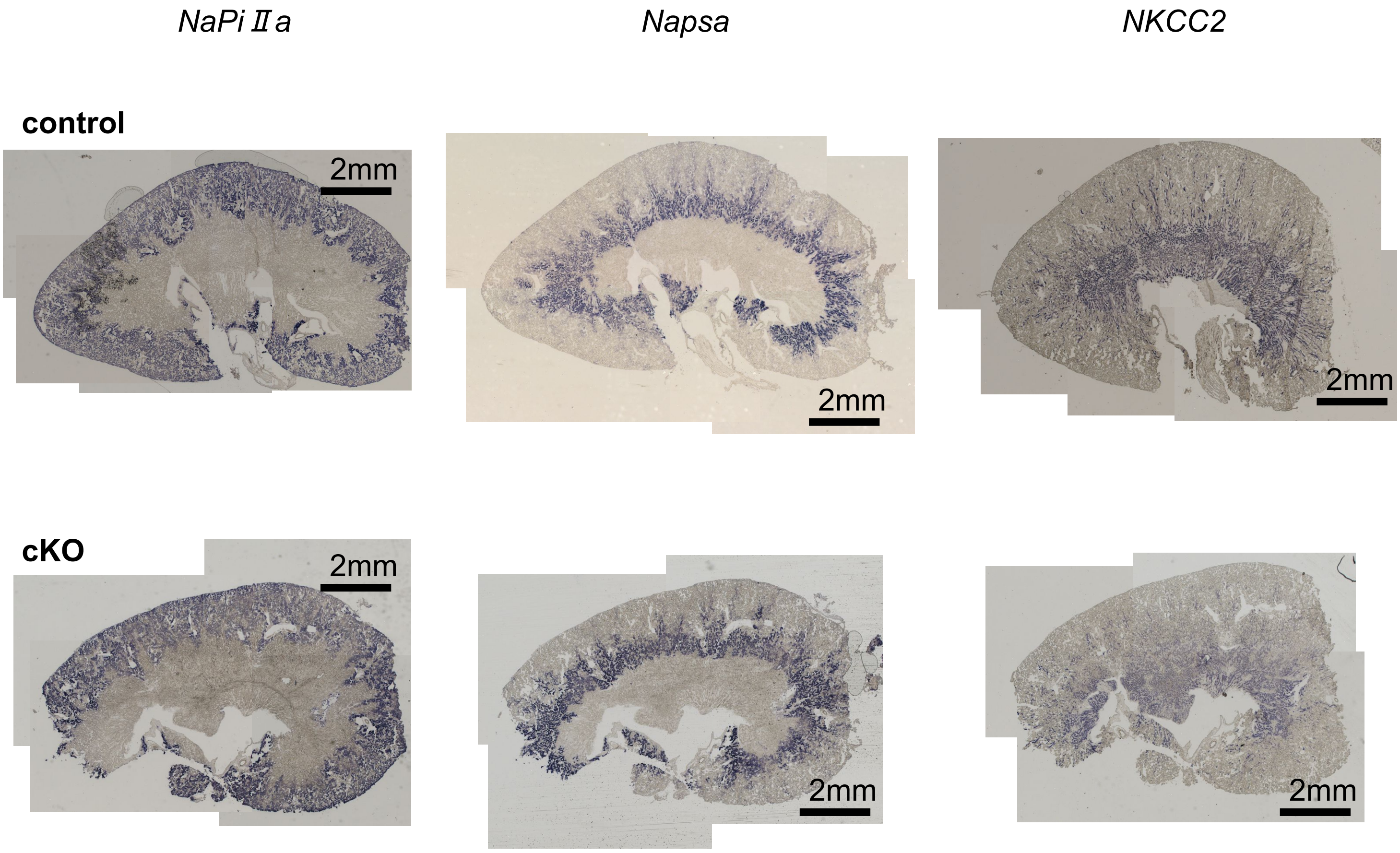

Fig. S5

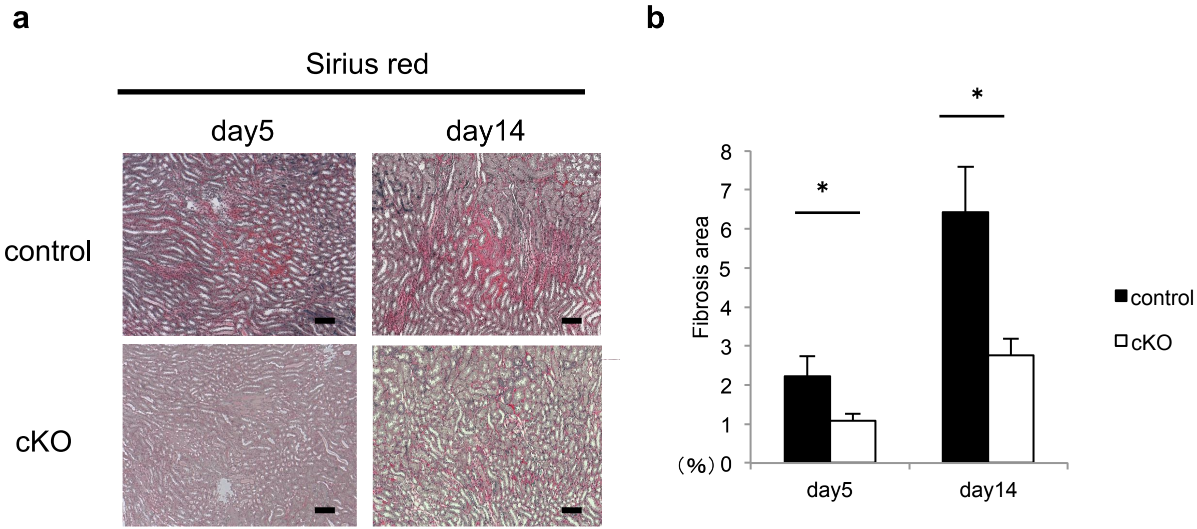

a
Sirius red
day5
day14
control
cKO
b
Fibrosis area
(%)
8
7
6
5
4
3
2
1
0
*
*
day5
day14
control
cKO

Fig. S6

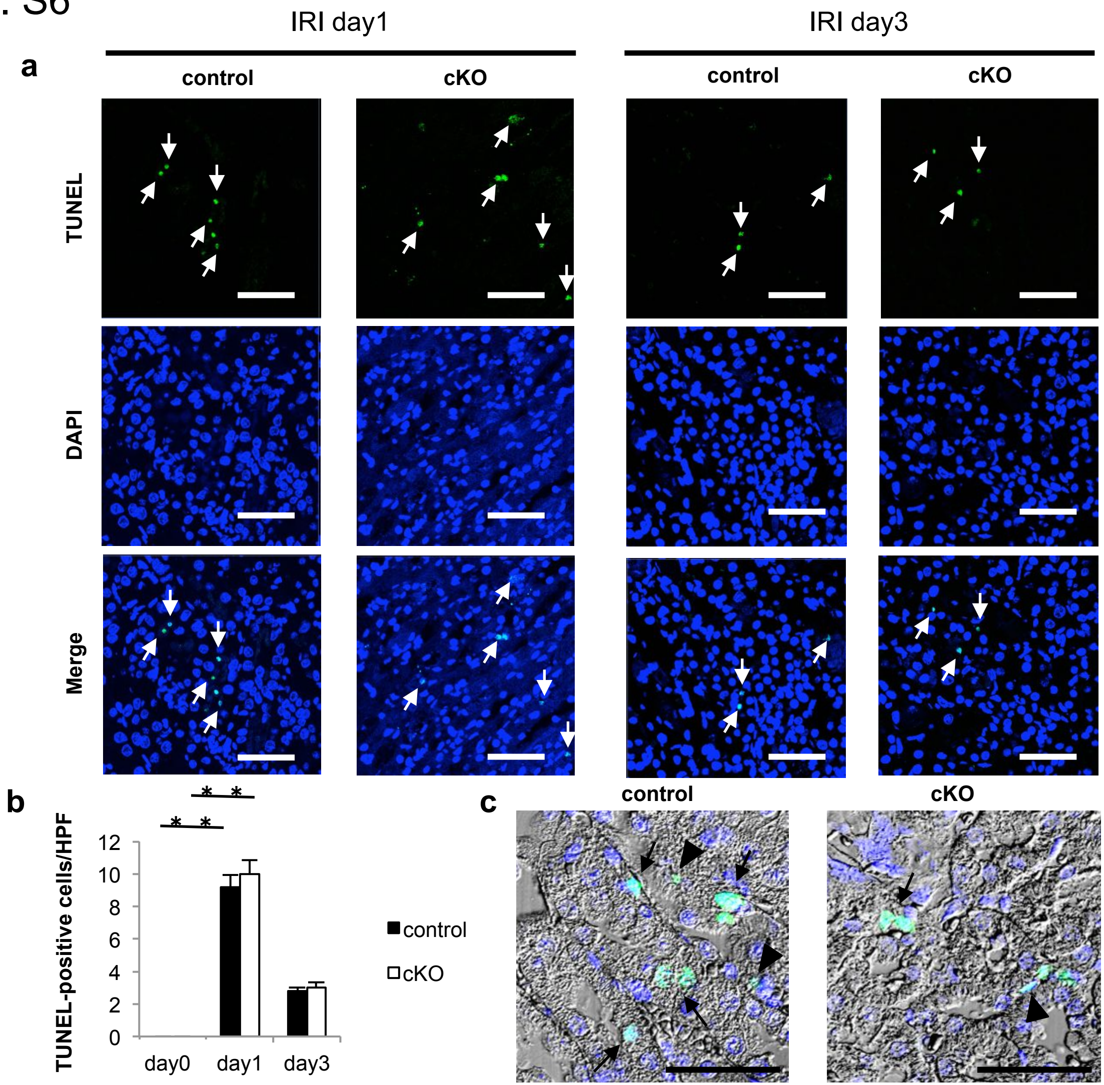

IRI day1
IRI day3
a
control
cKO
control
cKO
TUNEL
DAPI
Merge
b
* *
* *
TUNEL-positive cells/HPF
12
10
8
6
4
2
0
day0
day1
day3
control
cKO
c
control
cKO

Fig. S7

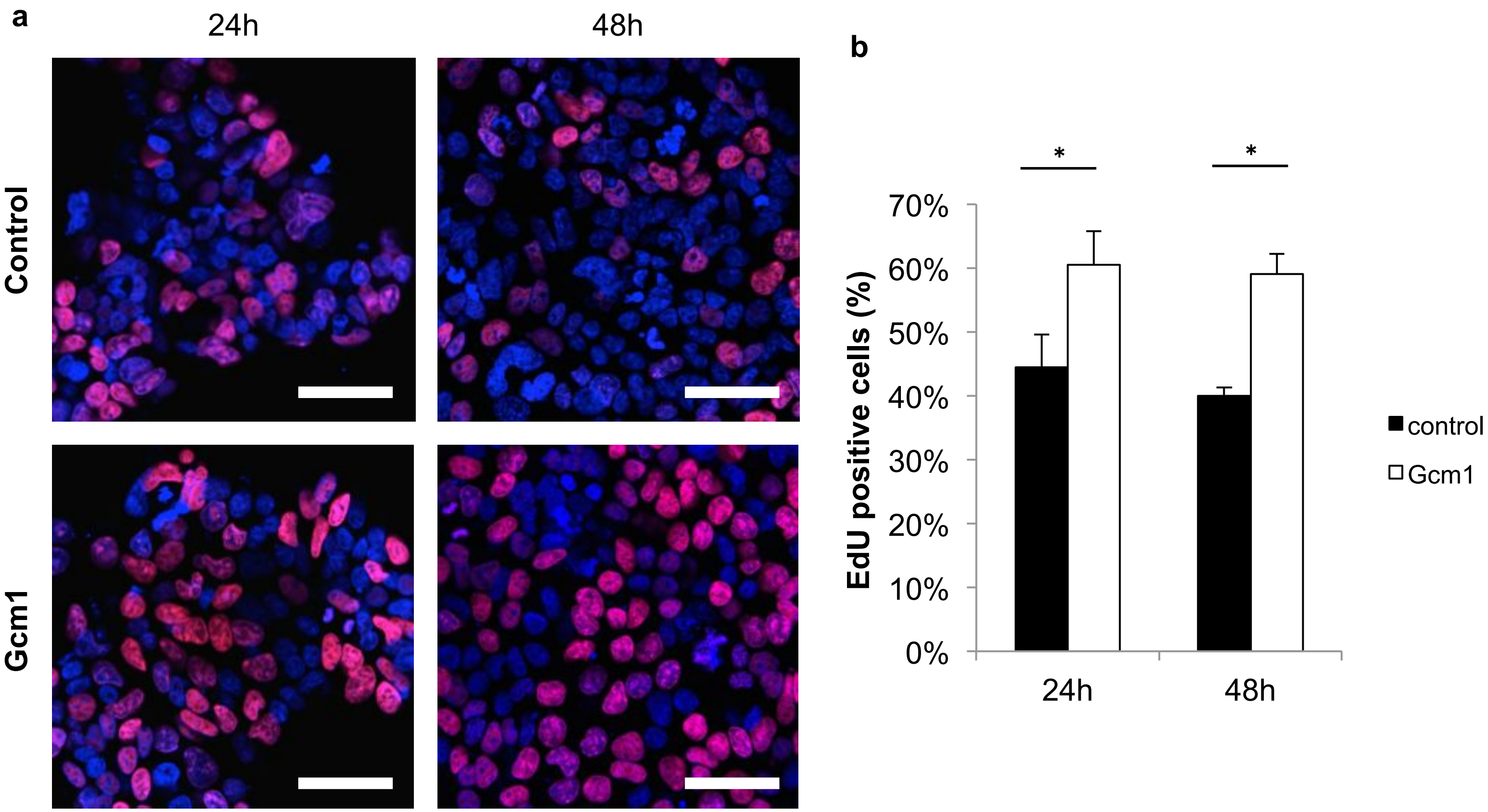

a
24h
48h
Control
Gcm1
b
*
*
70%
60%
50%
40%
30%
20%
10%
0%
EdU positive cells (%)
24h
48h
control
Gcm1

Fig. S8

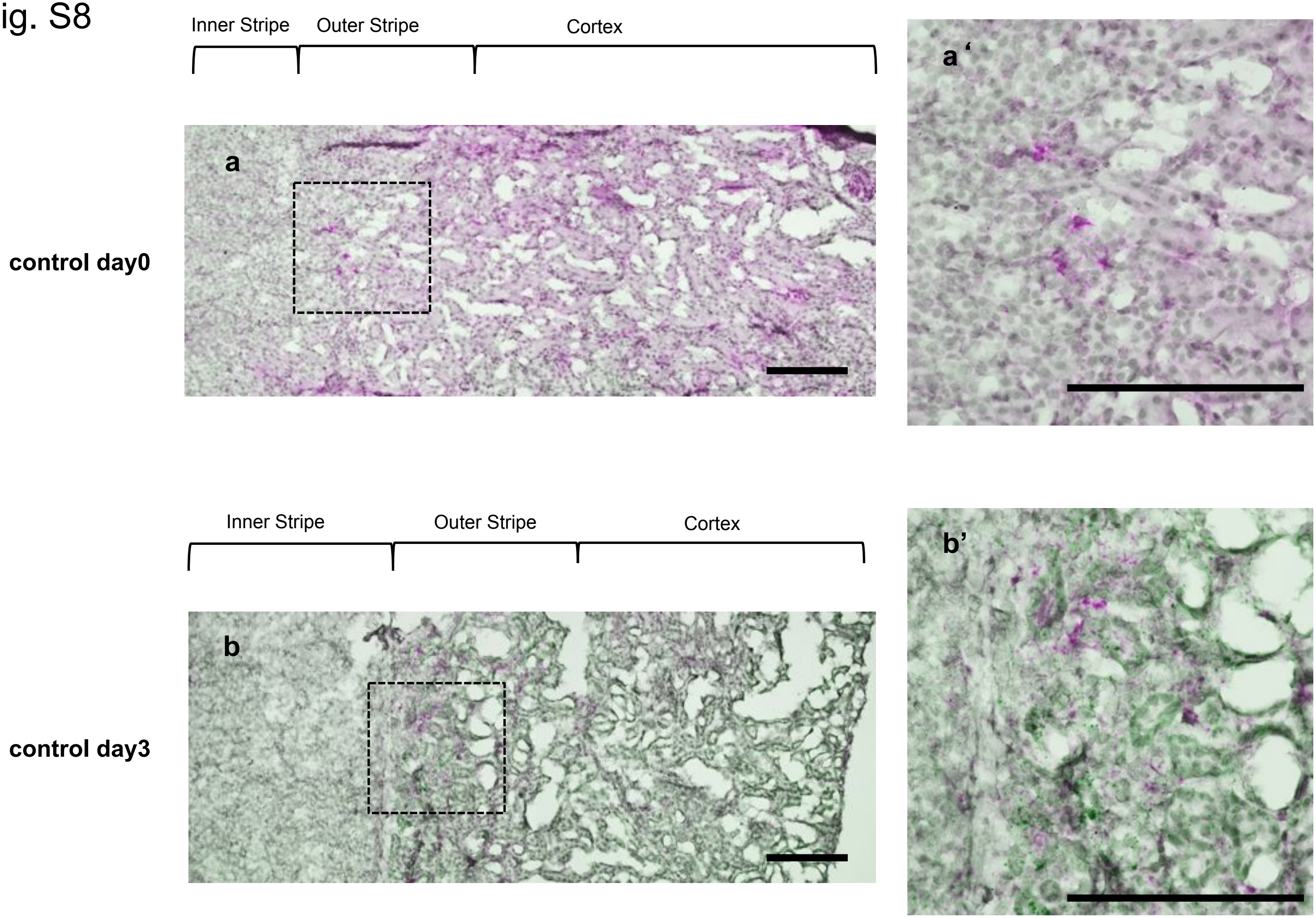
Inner Stripe
Outer Stripe
Cortex
control day0
a
a '
Inner Stripe
Outer Stripe
Cortex
control day3
b
b'

Fig. S9

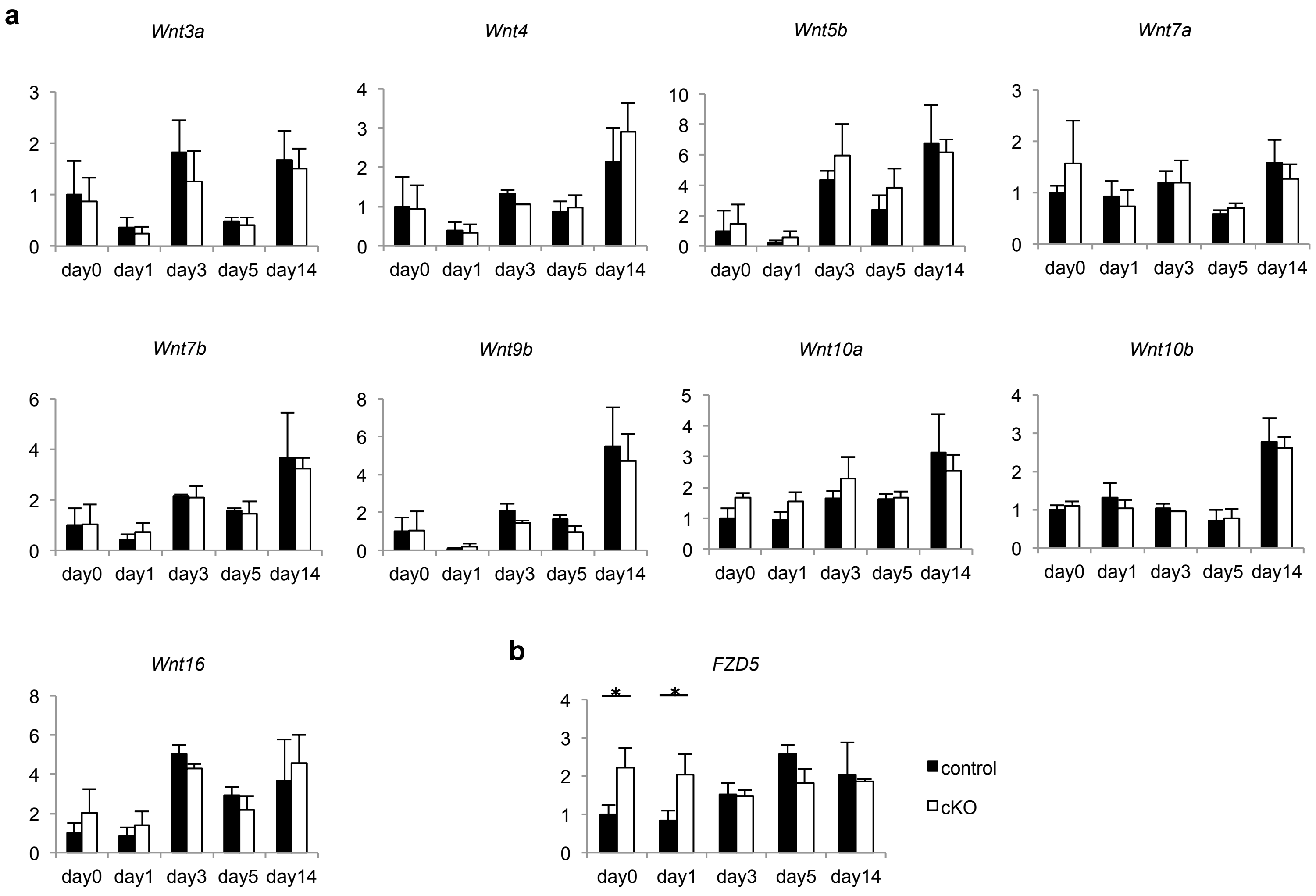
a
Wnt3a
Wnt4
Wnt5b
Wnt7a
Wnt7b
Wnt9b
Wnt10a
Wnt10b
Wnt16
b
FZD5
day0
day1
day3
day5
day14
*
control
cKO

Fig. S10

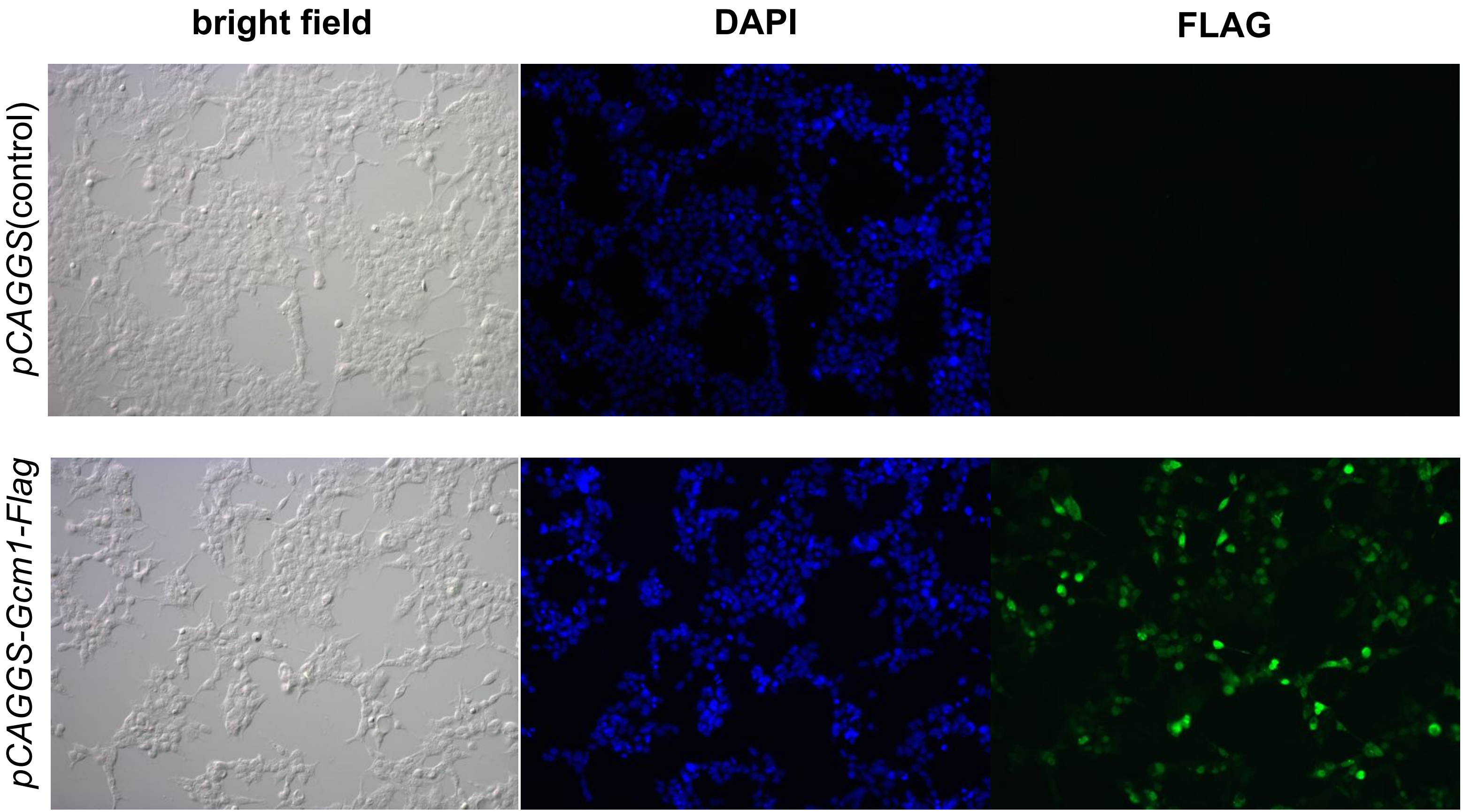

Table S1

	control	cKO
Number	10	10
sUN(mg/dL)	22.5 ± 0.15	23.5 ± 5.35
sCr(mg/dL)	<0.2	<0.2
sCa(mg/dL)	7.58 ± 0.36	8.32 ± 0.29
S-IP(mg/dL)	6.05 ± 0.73	6.87 ± 0.44
U-Ca(mg/dL)	<5.0	<5.0
U-IP(mg/dL)	160.4 ± 44.85	136.4 ± 58.87
U-TP(mg/dL)	292.9 ± 144.1	324.1 ± 62.96
U-Alb(μg/mL)	14.5 ± 2.3	16.2 ± 3.99
U-Glu(mg/dL)	8 ± 6.65	5 ± 2.68
U-Cr(mg/dL)	32.22 ± 5.92	26.76 ± 16.69

Date are x ±SD

Table S2

Gene name	Forward primer	Reverse primer	Size(bp)
qRT-PCR studies(mouse kidney)			
GAPDH	GGCAAATTCAACGGCACAGT	CGCTCCTGGAAGATGGTGAT	80
Gcm1	CCCGAGTTCTGAGACTCCAAAT	CGGGAGTTGTGGTTGTTGGT	280
HIF-1α	TCATCAGTTGCCACTTCCCCAC	CCGTCATCTGTTAGCACCATCAC	153
α-SMA	TCTCTATGCTAACAACGTCCTGTCA	CCACCGATCCAGACAGAGTACTT	153
Vimentin	CTTGAACGGAAAGTGGAATCCT	GTCAGGCTTGGAAACGTCC	135
Fibronectin	CGAGGTGACAGAGACCACAA	CTGGAGTCAAGCCAGACACA	149
MMP-7	TAGGCGGAGATGCTCACTTT	TTCTGAATGCCTGCAATGTC	150
Collagen 1	ATGCCGCGACCTCAAGATG	TGAGGCACAGACGGCTGAGTA	153
TGF-β1	GGAACTCTACCAGAAATATAGCAACAATTC	TGTATTCCGTCTCCTTGGTTCAG	141
FZD5	GCTTCAGAGGCCTGGAGTTT	GGGTAGTCATCCGAAGGCAG	146
qRT-PCR studies(HEK293)			
Human GAPDH	ACCACAGTCCATGCCATCAC	TCCACCACCCTGTTGCTGTA	452
Human TGF-β1	GGGTCTCCATCCCTGACGTT	CAGTGCCCAAGGTGCTCAAT	160
Human α-SMA	GGTGACGAAGCACAGAGCAA	CAGGGTGGGATGCTCTTCAG	150
primers used to synthesize RNA probes for the in situ hybridizaiton			
Gcm1	ACGACTGACTGGTTCCAGGAGTGGC	ATCCCACCTGTTGCTCTTGCCGG	1149
Napsa	CCTGAGCCAGGACAATCTGACT	CACATAGGGCCCCAAAAAGA	700
NKCC2	TGAGTTCGGTGGGTCAATAGG	CCATAGCCCTTTGCGAAGAA	855
NaPi2a(Slc34a1)	GCCAATGTCATCCAGAAGGT	CTCCTCGTTCCAGGCAGTAG	811
